# mRNA expression profile of serotonin receptor subtypes and distribution of serotonergic terminations in marmoset brain

**DOI:** 10.3389/fncir.2014.00052

**Published:** 2014-05-19

**Authors:** Rammohan Shukla, Akiya Watakabe, Tetsuo Yamamori

**Affiliations:** ^1^Division of Brain Biology, National Institute for Basic BiologyOkazaki, Japan; ^2^Department of Basic Biology, Graduate University for Advanced Studies (SOKENDAI)Okazaki, Japan

**Keywords:** mRNA expression, serotonin receptors, SERT, marmoset, mouse, comparison

## Abstract

To better understand serotonin function in the primate brain, we examined the mRNA expression patterns of all the 13 members of the serotonin receptor (*5HTR*) family, by *in situ* hybridization (ISH) and the distribution of serotonergic terminations by serotonin transporter (SERT) protein immunohistochemical analysis. Ten of the 13 *5HTR*s showed significant mRNA expressions in the marmoset brain. Our study shows several new features of the organization of serotonergic systems in the marmoset brain. (1) The thalamus expressed only a limited number of receptor subtypes compared with the cortex, hippocampus, and other subcortical regions. (2) In the cortex, there are layer-selective and area-selective mRNA expressions of *5HTR*s. (3) Highly localized mRNA expressions of *5HT1F* and *5HT3A* were observed. (4) There was a conspicuous overlap of the mRNA expressions of receptor subtypes known to have somatodendritic localization of receptor proteins with dense serotonergic terminations in the visual cortex, the central lateral (CL) nucleus of the thalamus, the presubiculum, and the medial mammillary nucleus of the hypothalamus. This suggests a high correlation between serotonin availability and receptor expression at these locations. (5) The *5HTR*s show differences in mRNA expression pattern between the marmoset and mouse cortices whereas the patterns of both the species were much similar in the hippocampus. We discuss the possible roles of *5HTR*s in the marmoset brain revealed by the analysis of their overall mRNA expression patterns.

## Introduction

Serotonin is an important neurotransmitter with multiple neuromodulatory functions in the central nervous sytem (CNS) (Millan et al., 2008; Lesch and Waider, 2012). Its receptors consist of 13 genetically, pharmacologically, and functionally distinct subtypes belonging to seven subfamilies (Alexander et al., 2011). All serotonergic receptors (*5HTR*s) are metabotropic G-coupled proteins except for *5HT3A*, which is ionotropic. Serotonergic innervations in mammalian CNS originate from the median and dorsal raphe nuclei of the mesencephalon (Moore et al., 1978; Bowker et al., 1983). Previous studies demonstrate that the termination patterns in mammalian subcortical regions are very similar across species (for thalamus see Lavoie and Parent, 1991 for basal ganglia see Lavoie and Parent, 1990 and Wallman et al., 2011). The difference in serotonin-dependent modulation among species therefore depends largely on the receptor type present in each locus.

To date, the distribution of serotonin and its receptors has been examined by immunohistochemical analysis, receptor ligand autoradiograpy, and *in situ* hybridization (ISH) in rodents (Mengod et al., 1996), nonhuman primates (Lidow et al., 1989; Hornung et al., 1990; Wilson and Molliver, 1991), and humans (Burnet et al., 1995; Raghanti et al., 2008). The detailed mRNA expression profiles of all the serotonin receptor genes in mice (Lein et al., 2007) and for some brain areas in human (Shen et al., 2012) are now publicly available in the Allen Brain Atlas (ABA) (ABA, 2009, 2012). Our previous study has shown that *5HT1B* and *5HT2A* are abundant in the visual cortex of macaque monkeys but not in rodents (Watakabe et al., 2009). This species difference demonstrates the importance of exploring the expression profiles of serotonin and its receptors in primates. In view of the heterogeneity of serotonin receptor subtypes, we wanted to obtain an integrated view of serotonergic modulation in primates by compiling the expression profiles of all the subtypes along with the termination pattern of serotonergic projections in the primate, which may contribute to an understanding of serotonin function in the primate brain.

For this purpose, we chose the common marmoset (*Callithrix jucchus*), a species of small New World monkey, that has attracted the interest of many biomedical researchers because of small size and ease of breeding (Mansfield, 2003). Moreover, the marmoset is the only nonhuman primate that can be used for generating germline-transmitted transgenic lines (Sasaki et al., 2009). In this study, we examined the mRNA expression profiles of all the known serotonin receptor subtypes by (1) ISH of *5HTR*s and (2) the serotonergic projection pattern by immunohistochemical analysis of the serotonin transporter (SERT) in various brain regions of the marmoset. Here, we discuss the differences and similarities of ISH patterns between some of the mouse and marmoset brain areas and publically available human data set by ABA (Shen et al., 2012).

Serotonergic terminations were particularly pronounced in the primary visual cortex (V1), the central lateral (CL) nucleus of the thalamus, the presubiculum, and the mammillary nucleus (MM) of the hypothalamus, where terminations overlapped with the abundant expressions of selected *5HTR* subtypes. Overall, when compared with mice, the serotonin receptor expression patterns in the marmoset brain were largely different in cortex but similar in hippocampus. The thalamus, which gates sensory information (Monckton and McCormick, 2002; Min, 2010), showed less receptor diversity than the cortex and hippocampus, which integrate sensory information.

## Materials and methods

### Ethics statement

All the experiments were conducted in accordance with the guidelines of the National Institutes of Health, and the Ministry of Education, Culture, Sports, Science and Technology (MEXT) of Japan, and were approved by the Animal Care and Use Committee in the National Institutes of Natural Sciences. We made all efforts to minimize the number of animals used and their suffering.

### Experimental animal, tissue preparation, and sectioning

Five brains of the adult common marmoset (*Callithrix jucchus*) (Two male: 2 years 6 months, and 3 years 5 months; Three female: ages-1 year 9 month, 2 years, and, 2 years 1 month) were used for confirmation of the mRNA expression patterns and their reproducibility. To avoid any chance of ambiguity owing to technical issues, the data presented in this paper are collected from the 6 years 2 months old, female marmoset monkey. We observed no individual difference in mRNA expression patterns. For tissue fixation, the animal was deeply anesthetized with Nembutal (100 mg/kg body weight, intraperitoneally) and perfused intracardially with saline (0.9% NaCl) and then with 4% paraformaldehyde in 0.1 M phosphate buffer. The brains were post-fixed for 5 h at room temperature and then cryoprotected with 30% sucrose in 0.1 M phosphate buffer at 4°C. The two hemispheres were sectioned separately, and approximately 600 coronal sections of 40 μm thickness encompassing the regions from the frontal cortex to the tectum were prepared from each hemisphere. All 13 serotonin receptor genes (Table 1) were examined for their expression patterns using an ISH technique. Two sets of tissue sections were immunohistochemically stained for SERT and nissl stained for laminar identification. For mice, data was collected from 3 male (46 weeks) and 2 female (42 and 35 weeks) B6 mice. The presubiculum, which showed expression of *5HT1F* (see results), could be best visualized by the sagittal sections of the mice brain, therefore we prepared sagittal sections of the mice brain. Because the visual (VIS), somatosensory (SS), and somatomotor (MO) areas cover the major part of the mouse brain and have analogous areas in the marmoset brain, these areas were selected for comparison between the mouse and marmoset brains.

**Table 1:** **Summary of ISH probes for 13 serotonin receptor genes, *HDC* and *GAD67* in the marmoset**.

**Gene**		**Primer**	**Ampilcon size (bp)**	**GC%**	**NCBI accession**
*5HT1A*	F:	TCCGACGTGACCTTCGGCTACC	703	61.02	XM_002744919
	R:	AGTTCCTGCTCCCCGATTCTCC			
*5HT1B*	F:	TATTGGCGCTCATCACCTTG	408	60.54	XM_002746745
	R:	TAGCCTGACGCCAGAAGAAG			
5HT1D	F:	ATCCCTGAATGCCACAGAAACC	917	56.92	XM_002750410
	R:	GGACCAAAGACACCACGAAGAA			
*5HT1E*	F:	TCACTCAGAAGAAATGCTGTGG	636	51.10	XM_001090686
	R:	TGAAAATGGAGATGGTCCAGAC			
*5HT1F*	F:	ACTTGACCTCAGAGGAACTGTT	987	42.93	XM_002761291
	R:	TGAGATACCCAAGCCATGTCAA			
*5HT2A*	F:	CTGGACCGCTACGTTGCCATCC	653	48.55	XM_002742676
	R:	CGATAGGTCTTGTTGAACAGTG			
*5HT2C*	F:	CCACTACCTAGATATTTGTGCC	754	44.97	XM_002763170
	R:	TGTACACCAGAGGATTGATTCC			
*5HT3A*	F:	AGTACTGGACTGATGAGTTTC	683	51.83	XM_002754423
	R:	CAGAGCCATGCACACCACAAA			
*5HT3B*	F:	GGGAATTCTAGCCACAGATACG	785	47.13	XM_002754430
	R:	CCAGCACACTGGTCTTGAACAC			
*5HT4*	F:	AGAAGGTCGTGCTGCTCACGTT	816	49.26	XM_002744348
	R:	GGACAGTGTAGTCTATGAAAGG			
5HT5	F:	TGCTGGTGCTGGCTACCATCCT	604	63.41	XM_002751806
	R:	ATGAGGATGCCCACCATGAGGG			
*5HT6*	F:	CAACTTCTTCCTGGTGTCGCTC	803	65.88	XM_002750377
	R:	GCTTGAAGTCCCGCATGAAGAG			
*5HT7*	F:	GGCAGAATGGGAAATGTATGGC	655	50.84	XM_002756389
	R:	GAGAGCTTCCGGTTGATATTCC			
*HDC*	F:	TGATGGAGCCTGAGGAGTACAG	741	55.47	XM_002753473
	R:	TGGTCCCTAGTGTTGCACAGAC			
*GAD67*	F:	GCTTCTTGCAAAGGACCAAC	858	49.10	XM_002749363
	R:	CCTTCTGTTTGGCTTCAAGA			

Note that owing to unavailability of the marmoset-specific 5HT1E sequence in the public database, the 5HT1E primers were designed using the macaque 5HT1E sequence. The hybridization temperature for 5HT3A was 65°C and that for others was 60°C. The amplicon includes the primer sequence. F indicates forward and R indicates reverse.

### ISH

Both the sense and antisense digoxigenin (DIG)-labeled riboprobes used in this study were prepared from plasmids containing PCR-amplified fragments of marmoset *5HTR*s, histidine decarboxylase (*HDC*) and *GAD67* genes. For *VgluT1*, riboprobes previously used for monkey ISH were used (Komatsu et al., 2005). To confirm the specificity of the antisense probes, the sense probes were used as the control in all the experiments. Details of the probes designed for the marmoset are shown in Table 1 and those for the mouse are shown in Table S1. Single and double-colored ISH were performed using the methods described in the papers of our group (Watakabe et al., 2007, 2009; Takaji et al., 2009). Briefly, free-floating sections were treated with proteinase K (5 μg/mL) for 30 min at 37°C, acetylated, then incubated in a hybridization buffer [5X SSC, 2% blocking regent (Roche Diagnostics, Basel, Switzerland), 50% formamide, 0.1% N-lauroylsarcosine, 0.1% SDS] containing 0.5 μg/mL DIG-labeled riboprobes at 65°C for *5HT3A* receptor gene and 60°C for the others. The sections were sequentially treated in 2XSSC/50% formamide/0.1% N-lauroylsarcosine for 15 min at 60°C twice, 30 min at 37°C in RNase buffer [10 mM Tris-HCl (pH 8.0), 1 mM ethylenediaminetetraacetic acid (EDTA), 500 mM NaCl] containing 20 μg/mL RNase A (Sigma Aldrich, Saint Louis, MI), 15 min at 37°C in 2XSSC/0.1% N-lauroylsarcosine twice, and 15 min at 37°C in 0.23 SSC/0.1% N-lauroylsarcosine twice. The hybridization probe was detected with an alkaline-phosphatase conjugated anti-DIG antibody using DIG nucleic acid detection kit (Roche Diagnostics).

For double-colored ISH, the sections were cut to 15 or 20 μm thickness. The hybridization and washing were carried out as described above, except that both DIG- and fluorescein-labeled probes were used for the hybridization. After blocking in 1% blocking buffer (Roche Diagnostics) for 1 h, the probes were detected in two different ways. For the detection of fluorescein probes, the sections were incubated with an anti-fluorescein antibody conjugated with horseradish peroxidase (Jackson ImmunoResearch Laboratories, West Grove, PA: #200-032-037, 1:4000 in the blocking buffer) for 3 h at room temperature. After washing in TNT buffer [0.1 M Tris-HCl (pH 7.5), 0.15 M NaCl, 0.1% Tween20] 3 times for 15 min, the sections were treated with 1:100 diluted TSA-Plus reagents (Perkin Elmer, Boston, MA) for 30 min following the manufacturer's instruction, and the fluorescein signals were converted to dinitrophenol (DNP) signals. After washing with TNT buffer 3 times for 10 min, the sections were incubated overnight at 4°C with an anti-DNP antibody conjugated with Alexa 488 (1:500, Molecular Probes, Life Technologies Corporation, Carlsbad, CA) in 1% blocking buffer for the fluorescence detection of the DNP signals. At this point, an anti-DIG antibody conjugated with alkaline phosphatase (1:1000, Roche Diagnostics) was also incubated for the detection of the DIG probes. The sections were washed 3 times in TNT buffer, once in TS 8.0 [0.1 M Tris-HCl (pH 8.0), 0.1 M NaCl, 50 mM MgCl_2_], and the alkaline phosphatase activity was detected using HNPP fluorescence detection kit (Roche Diagnostics) following the manufacturer's instruction. This substrate was incubated for 30 min and the incubation was stopped in PBS containing 10 mM EDTA.

### SERT immunohistochemistry

Immunohistochemical analysis was conducted essentially in accordance with the protocol previously reported (Sakata et al., 2002). Briefly, we used antisera raised against SERT (1:12000) as primary antibodies and biotinylated goat anti-rabbit IgG (1:1000) as secondary antibodies (all supplied by Immunostar, Inc., USA). The free-floating sections were incubated consecutively in PBS containing 1% H_2_O_2_ for 10 min at room temperature, and then in PBS with 0.2% Triton X-100 (PBST) and 5% normal goat serum (serum of the species of the secondary antibody) for 60 min at room temperature. This was followed by overnight incubation in a buffer containing 1% normal goat serum and the primary antibody at 4°C. After incubation with the biotinylated secondary antiserum for 2 h at room temperature, the sections were processed with an avidin-biotinylated horseradish peroxidase complex (1:200; Vectastain ABC Elite kit, Vector Laboratories, Burlingame, CA, USA) in PBST at room temperature for 1 h and the immunoreaction was visualized by staining with nickel-enhanced coloring solution (0.2 mg/mL diaminobenzidine: DAB, 0.03% H_2_O_2_, 0.03% nickel chloride in TBS).

### Data quantification

Representative areas and regions were identified by referring to the stereotaxic atlas of the marmoset brain (Palazzi and Bordier, 2008; Yuasa et al., 2010; Paxinos et al., 2011) and Nissl staining. The intensity of hybridization signals of different genes varied across different areas of the brain. We present the intensity of the signals as mRNA expression level rated as very low (+), low (++), moderately high (+++), or high (++++) by visual inspection (Tables 2, 3). To show the weak signals, the images were adjusted to different contrast levels. In some instances, this enhanced the noise from the adjacent white matter. The true signals based on size and color can be clearly differentiated from the noise (see Figures S8A–D). Because DIG based ISH provides cellular resolution, we also distinguished dense and disperse expression profiles for relevant regions. To provide a more objective comparison of the laminar distribution of expression between the mouse and marmoset cortices, we analyzed the optical densities of ISH signals using imageJ image analysis software (Abramoff et al., 2004) (Figures S7A–C). After making the contrast level the same for all images of the same gene, individual images were inverted and optical density was measured using the straight line tool that sampled all layers of the cortex. To subtract the background noise, the optical density of either layer I or white matter (the region where there was no expression above background level) was taken as the control.

**Table 2:** **Arbitrary values assigned for different levels of expression in cortical brain areas**.

		***5HT1A***	***5HT1B***	***5HT1E***	***5HT1F***	***5HT2A***	***5HT2C***	***5HT3A***	***5HT4***	***5HT6***	***5HT7***
**Area46**	1:	−	−	−	−	−	−	−	−	−	−
	2:	++++	±	++	−	±	+++^vs^	−	+++	+++	±
	3:	+++	±	++^s^	−	++++	−	−	++^s^	+++	±
	4:	+++	±	+^s^	−	++++	−	−	++^s^	+++	±
	5:	+++	+	++^s^	−	+++	++++^vs^	−	++^s^	+++	±
	6:	++	−	+^s^	−	+	−	−	+	++	±
**Area6**	1:	−	−	−	−	−	−	−	−	−	−
	2:	++++	±	++	−	±	++^vs^	−	++^s^	+++	±
	3:	+++	±	+	−	+++	−	−	+	++	±
	4:	+++	±	+	−	+++	−	−	+	++	±
	5:	++	+	+	−	++	++++^vs^	−	+	++	±
	6:	+	−	+	−	+	−	−	±	+	±
**M1**	1:	−	−	−	−	−	−	−	−	−	−
	2:	++++	±	++	−	±	++^s^	−	++^s^	+++	±
	3:	++	±	±	−	+++	−	−	+^s^	++	±
	4:	+	±	±	−	+++	−	−	++^s^	+	±
	5:	+	++	±	−	++	++++^vs^	−	±	+	±
	6:	+	−	±	−	+	−	−	±	+	±
**S1**	1:	−	−	−	−	−	−	−	−	−	−
	2:	++++	±	++	−	±	++^s^	−	++^s^	+++	±
	3:	+	±	+	−	++++	−	−	+^s^	+	±
	4:	±	±	++^s^	−	++++	−	−	++^s^	±	±
	5:	±	±	++^s^	−	++	+++^vs^	−	±	±	±
	6:	±	±	+	−	+	−	−	±	±	±
**MT**	1:	−	−	−	−	−	−	−	−	−	−
	2:	++++	±	+++	−	±	++^s^	−	++^s^	+++	±
	3:	++	±	++	−	+++	−	−	+^s^	++	±
	4:	±	±	+	−	+++	−	−	+	±	±
	5:	+	±	++^s^	−	++	+++^vs^	−	±	+	±
	6:	+	±	++^s^	−	+	−	−	±	+	±
**ITG**	1:	−	−	−	−	−	−	−	−	−	−
	2:	++++	±	+++	−	+	+++^vs^	−	+++	+++	±
	3:	+++	±	++^s^	−	++	−	−	+++^s^	+++	±
	4:	+	±	±	−	+++	−	−	+	+	±
	5:	+++	±	++^s^	−	+++	+++^vs^	−	+++	+++	++
	6:	++	+	±	−	+	−	−	±	±	±
**TE**	1:	−	−	−	−	−	−	−	−	−	−
	2:	++++	±	++	−	+	++^vs^	−	+++	+++	±
	3:	+++	±	+^s^	−	+++	−	−	+++^s^	+++	±
	4:	+	±	±	−	++++	−	−	+	+	±
	5:	+++	+	++^s^	−	+++	+++^vs^	−	+++	+++	+
	6:	+++	+	±	−	+	−	−	±	±	±
**V1**	1:	−	−	−	−	−	−	−	−	−	−
	2:	++++	+++	+++	−	++	−	−	+++	+++	±
	3:	+++	+++	++	−	+++	−	−	+++^s^	++	±
	4:	++	++++	±	−	>++++	−	−	+	±	±
	5:	±	−	++^s^	−	+	+++^vs^	−	+++^s^	++^s^	±
	6:	±	−	+^s^	+++	+	−	−	±	++^s^	±
**V2**	1:	−	−	−	−	−	−	−	−	−	−
	2:	++++	++	++	−	++	++^vs^	−	+++	+++	±
	3:	++	+	+^s^	−	+++	−	−	+++	++	±
	4:	+	+	+^s^	−	++	−	−	+	±	±
	5:	±	−	+^s^	−	++	+++^vs^	−	++	+^s^	±
	6:	±	−	+^s^	−	+	−	−	±	+^s^	±
**CG**	1:	−	−	−	−	−	−	−	−	−	−
	2:	++++	−	++	−	±	++^vs^	−	++	+++	−
	3:	+	−	+^s^	−	+++	−	−	+++^s^	++	−
	5:	±	++	+^s^	−	+++	+++^vs^	−	++	+++^s^	+
	6:	±	−	+^s^	−	±	−	−	±	±	−
**ER**	1:	−	−	−	−	−	++	−	−	−	−
	2:	++++	++	+	−	++++	±	−	++	++++	+++
	3:	+++	+	+	−	−	+++	−	+++^s^	+++	++
	5:	+++	+	+++	−	+++	−	−	+++^s^	++	+
	6:	+++	+++	±	−	+++	−	−	±	±	+++

++++, high; +++, moderately high; ++, low; and +, very low levels of expression. ± was assigned to areas of uncertain level of expression. The superscripts “S” and “VS” denote sparse and very sparse expressions, respectively. The numbers from 1 to 6 denote the layers of the cortex. The abbreviations of the cortical areas are the same as those mentioned in the main text.

**Table 3:** **Arbitrary values assigned for different levels of expression in subcortical brain areas**.

**Area**	***5HT1A***	***5HT1B***	***5HT1E***	***5HT1F***	***5HT2A***	***5HT2C***	***5HT3A***	***5HT4***	***5HT6***	***5HT7***	**Figure References**
**THALAMUS**											
**Ventral anterior (VA)**	+++	++	−	−	−	−	−	−	++	+++	7, S2, S4
**MEDIAL GROUP**											
Mediodorsal nucleus (MD)	+	+++	−	−	±	++	−	−	++	++	7
Central lateral nucleus (CL)	+++	±	++	−	+++	+++	−	−	++	++	7
**VENTRAL LATERAL GROUP**											
lateral dorsal nucleus (LD)	+	+++	−	−	−	−	−	−	+++	+++	7
Ventral lateral nucleus (VL)	+	+++	−	−	−	−	−	−	+	+++	7
**VENTRAL POSTERIOR GROUP**											
Ventral posterior lateral (VPL)	+	+++	−	−	−	−	−	−	+	+++	S2, S4
Ventral posterior medial (VPM)	+	+++	−	−	−	−	−	−	+	+++	S2, S4
**POSTERIOR GROUP**											
Medial geniculate body (MG)	+	+++	±	−	−	−	−	−	++	++	S3
Lateral geniculate body (LG)	+	++++	−	−	−	−	−	−	+	++	S4
Pulvinar	++	+++	−	−	−	−	−	−	++	+++	S2
Thalamic reticular nucleus (Rt)	+	+++	−	−	+++	+++	−	−	−	−	S2
**HIPPOCAMPUS**											
CA1	++++	++	+++	−	±	++++^s^	++++^s^	++	+++	++	6, S2
CA2	++++	+++	++++	+	±	++++^s^	++++^s^	+++	++++		6, S2
CA3	++++	++	+++	−	±	++++^s^	++++^s^	++	+++	++	6, S2
Dentate gyrus	++++	++	+++	−	++++	−	−	+++	+++	±	6, S2
Subicular complex	++	++	++	+++	+	++^s^	−	++	++	++	6, S2
**AMYGDALA**											
Basolateral(BLa)	+++	+	++	−	+	++++^s^	−	+	+	+	10
Basomedial(BMa)	+++	+	++	−	+	++++^s^	−	+	+	+	10
Cortical amygdaloid (Co)	+++	+	++	−	+	++++^s^	−	++	++	++	10
Medial amygdaloid (Me)	+++	+	++	−	+	++++	−	+	+	+	10
Lateral amygdaloid (La)	++	+	++	−	+++	++++^s^	−	+	+	±	10
**HYPOTHALAMUS**											
Medial mammillary nucleus (MM)	±	−	±	−	++++	−	−	−	++	+++	8, S2
Lateral mammillary nucleus (ML)	+++	++	−	−	−	++++	−	−	++	−	8, S2
Ventral tuberomammillary (VTM)	−	−	−	++++	−	−	−	−	±	+++	8, S2
**DORSAL STRIATUM**											
Putamen	++	++	+	−	+	++++	−	++++	+++	+	12
Caudate nucleus	++	++	+	−	+	++++	−	++++	+++	+	12
Medial septum	+++	±	−	−	+++	−	−	±	±	±	12
Lateral septum	+++	+++	++	−	−	+++	−	+	+	++	12
**VENTRAL STRIATUM**											
Globus pallidus internal (IGP)	+	−	++	−	++	++++	−	+	++	−	S2
Globus pallidus external (EGP)	+	−	++	−	++	++	−	+	++	−	S2
Substantia nigra reticulata (SNr)	++	++	+++	−	±	+++	−	+++	+++	+++	13
Substantia nigra compacta (SNc)	++	++	+++	−	+	+++	−	+++	+++	+++	13
**MIDBRAIN TECTUM**											
Superior colliculi (SC)	++++	++	−	−	+	+++	−	−	+++	++	11

++++, high; +++, moderately high; ++, low; +, very low levels of expression. ± was assigned to areas of uncertain level of expression. The superscript “S” denotes sparse expression.

## Results

We examined the mRNA expression patterns of all 13 known serotonin receptor subtypes. We found significant expressions of 10 of them; we were unable to detect the expressions of *5HT1D*, *5HT3B*, and *5HT5A* mRNAs in the marmoset brains examined. *5HT3A* mRNA was exclusively expressed in the CA fields of the hippocampus. *5HT1F* mRNA was expressed only in layer VI of V1, the presubiculum, and the lateral mammillary body (LM) of the hypothalamus. In general, the expression patterns of all the genes differed in both the intensity and density of ISH signals throughout the marmoset brain. Most of the examined nuclei showed overlapping expressions of multiple *5HTR* subtypes. In the cerebral cortex, most subtypes of *5HTR* were expressed, whereas we found only limited *5HTR* subtypes in the thalamus. The termination pattern obtained by SERT immunohistochemical analysis in our study was similar to those obtained in previous studies of marmosets (Hornung et al., 1990; Hornung and Celio, 1992) and squirrel monkeys (Lavoie and Parent, 1991). Below, we first describe the patterns of expression of *5HTR* mRNAs, across cortical areas. We then describe their expression patterns in the hippocampus, thalamus, superior colliculus, hypothalamus, amygdala, striatum, and substantia nigra. We also compared anti-SERT immunoreactivity with *5HTR* mRNA expression profiles.

### Serotonin receptor mRNA expression in cortical areas

**Figure 1:**
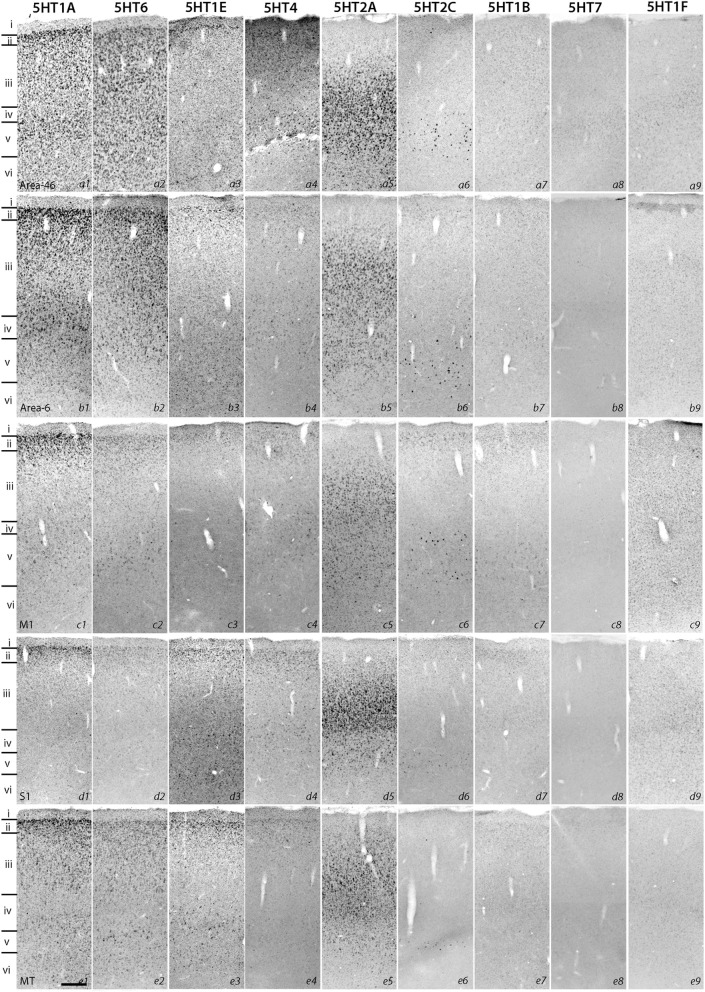
**ISH expression profiles of *5HTRs* in cortex**. Area 46, area 6, primary motor cortex (M1), primary somatosensory cortex (S1), and V5 (MT). Layers identified by Nissl staining (not shown) are indicated on the left. Note that all images of a given gene are grouped together and presented at the same contrast level. Scale bar: 100 μm.

To examine the expression profiles in the association and sensory areas of different lobes of the cortex in the rostrocaudal axis, we examined areas 46 and 6, the primary motor cortex (M1), the primary somatosensory cortex (S1), the inferotemporal gyrus (ITG), area V5 (MT), the temporal cortex (TE), the primary visual cortex (V1), and the secondary visual cortex (V2). Besides these six-layered areas, we also examined the cingulate (CG) cortex and entorhinal cortex (Er) of four-layered areas. In these cortical areas, nine of the ten serotonin receptor genes (i.e. excluding *5HT3A*) were expressed. We noted that several *5HTR* subtypes exhibited gradients in expression profiles in the sensory and association areas. The most conspicuous example was the V1-V2 border (Figures 3A–F), which has the most differentiated architecture of the primate cortex. *5HT2A*, a gene abundantly expressed in the middle layer, also showed a marked difference in mRNA expression level between S1 and M1 (Figure 1, c5, d5).

**Figure 2:**
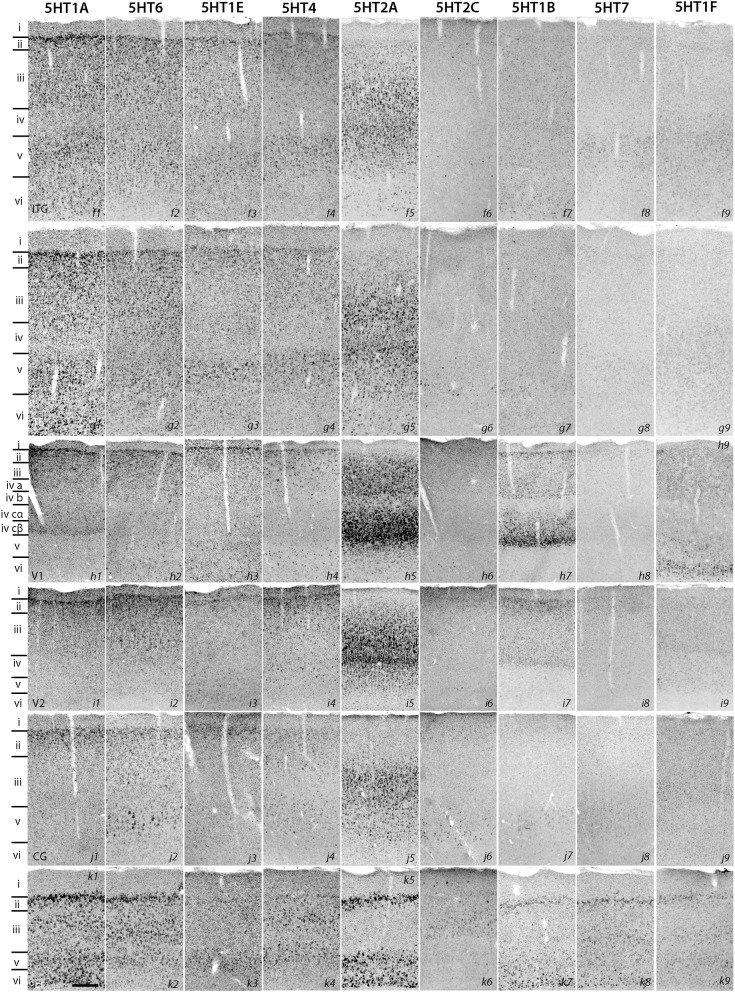
**ISH expression profiles of *5HTRs* in cortex**. Inferotemporal gyrus (ITG), temporal cortex (TE), primary visual cortex (V1), secondary visual cortex (V2), cingulate cortex (CG), and entorhinal cortex (Er). Layers identified by Nissl staining (not shown) are indicated on the left. Note that all images of a given gene are grouped together and presented at the same contrast level. Scale bar: 100 μm.

Despite such differences in mRNA expression level between areas, a few *5HTR* subtypes exhibited similarities in their laminar expressions across areas when compared with their expression in the upper, middle, and lower layers. In addition, a few *5HTR*s showed sporadic expression across the cortex. *5HT1A*, *5HT6*, *5HT1E*, and *5HT4* were all generally expressed in the upper layers irrespective of the area (Figures 1, 2, see a1–4 to k1–4). This group of genes shared several similar characteristic features in their expression profiles. Compared with *5HT1A* and *5HT6*, both *5HT1E* and *5HT4* were less abundant in layer II. To test our hypothesis of dense expression in excitatory neurons and sparse expression in inhibitory neurons we performed the double hybridization of *5HT1A*, *5HT1E*, *5HT4*, and *5HT6* using excitatory (*VgluT1*) and inhibitory (*GAD67*) neuronal markers in V1. Indeed, our results indicated the presence of *5HT1A* and *5HT6* in excitatory neurons and that of *5HT4* in inhibitory neurons (Figure 4). We were unable to obtain signals for *5HT1E* using either of the markers. In the frontal (areas 46 and 6) and temporal (ITG and TE) association areas, *5HT1A* and *5HT6* were expressed from layers II through V, but their mRNA expression levels in layer IV of ITG and TE were much lower. In contrast to the wide-spread expression in the association areas, in early sensory areas, such as S1, V1, and V2, their expression was mostly limited to layer II. The area difference was conspicuous for *5HT1A* and *5HT6* but not for *5HT1E* and *5HT4*.

*5HT2A* mRNA was expressed at various levels from layers III to V throughout the neocortical areas. Its expression was more abundant in lower tiers of layer III and relatively sparse in layers IV and V. *5HT2C* was expressed sparsely in layers II and V. Although *5HT2A* and *5HT2C* expressions overlapped in layer V, they generally exhibited opposite patterns of layer and area distributions: *5HT2A* was highly expressed in V1 whereas *5HT2C* showed a gradient in expression from being rostrally high to caudally low and was almost undetectable in V1 and V2. In the entorhinal cortex, both the genes were expressed complementarily; unlike in other areas, *5HT2A* was present in layer II and lower layers V and VI (Figure 2, k5), whereas *5HT2C* was expressed in layers I and III (Figure 2, k6) where *5HT2A* was little expressed. We performed double hybridization of *5HT2A* with *GAD67* and *VgluT1* neuronal markers in V1. Because the expression of *5HT2C* was scant in V1, we performed its double hybridization in sections from the frontal cortex and observed layer V encompassing all areas of the frontal cortex covered in the section. *5HT2A* was mainly expressed in *VgluT1*-positive excitatory neurons (Figure 5), and almost all the cells expressing *5HT2C* were positive for *GAD67* inhibitory neurons (Figure 5).

**Figure 3:**
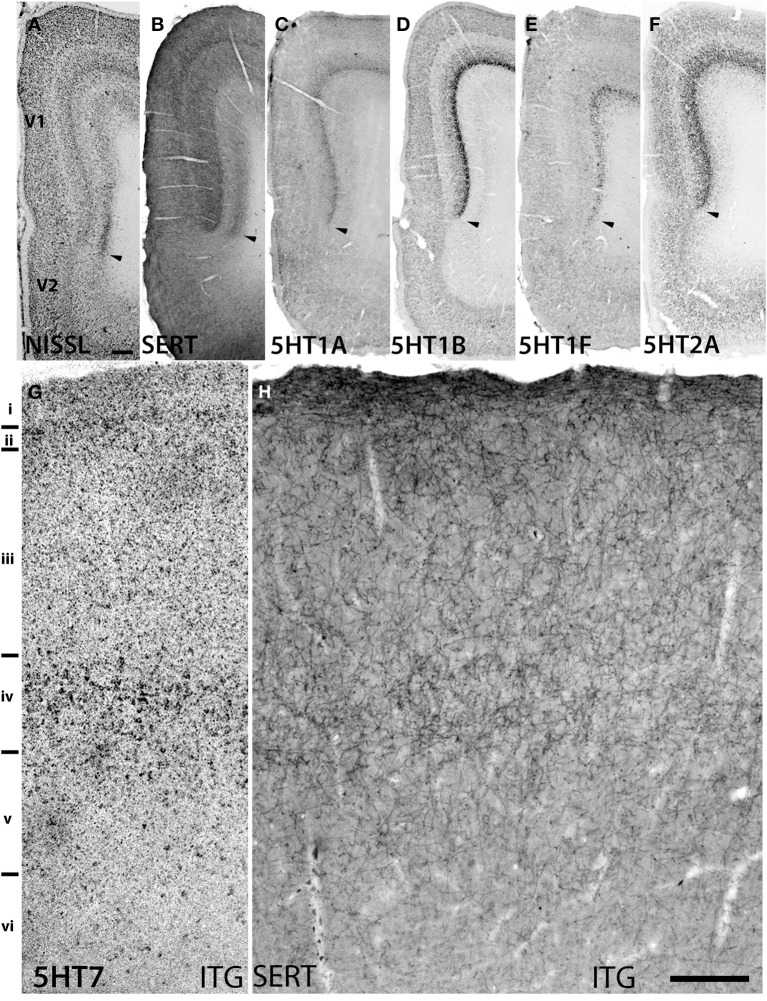
**Sections showing specific staining at V1 and V1-V2 border (A–F) and ITG (G,H)**. **(A)** Nissl staining and architecture of V1-V2. **(B)** Immunohistochemical staining with anti-SERT antibodies. Note that the projection density is particularly high in layers IV and VI. **(C**–**F)** Expression profiles of *5HT1B*, *5HT2A*, *5HT1A*, and *5HT1F*. The arrow heads indicate the border between V1 and V2. **(G,H)** show the overlap of increased expression of *5HT7* in ITG with serotonergic projections at layer IV. The precise layers of expression of the genes studied here can be seen in Figure 2. Each image has been adjusted at a contrast level that shows the clearest border. Scale bars for **(A**–**F)**, 200 μm and for **(G,H)**, 100 μm.

The expression levels of *5HT1B*, *5HT1F*, and *5HT7* mRNAs were low throughout the neocortical areas. However, *5HT1B* mRNA was abundantly expressed in V1 (Figures 2, h7 and 3D) and significantly in V2 (Figure 2, i7); a higher intensity of *5HT1F* mRNA signals was observed in layer VI of V1 (Figures 2, h9 and 3E) and *5HT7* mRNA was expressed at a moderately high level in layer IV of area ITG (Figure 2, f8). Note that the increase in the expression level of *5HT7* overlapped with the enhanced serotonergic terminations at ITG (Figure 3G). *5HT1B* was also sparsely expressed in layer V of M1 (Figure 1, c7) and CG (Figure 2, j7). In the entorhinal cortex, *5HT1B* and *5HT7* showed similar expression patterns, that is, highly expressed in layer II and moderately expressed in lower layers.

### Marmoset V1 is characterized by serotonergic projections and expression of a group of 5HTR subtypes

**Figure 4:**
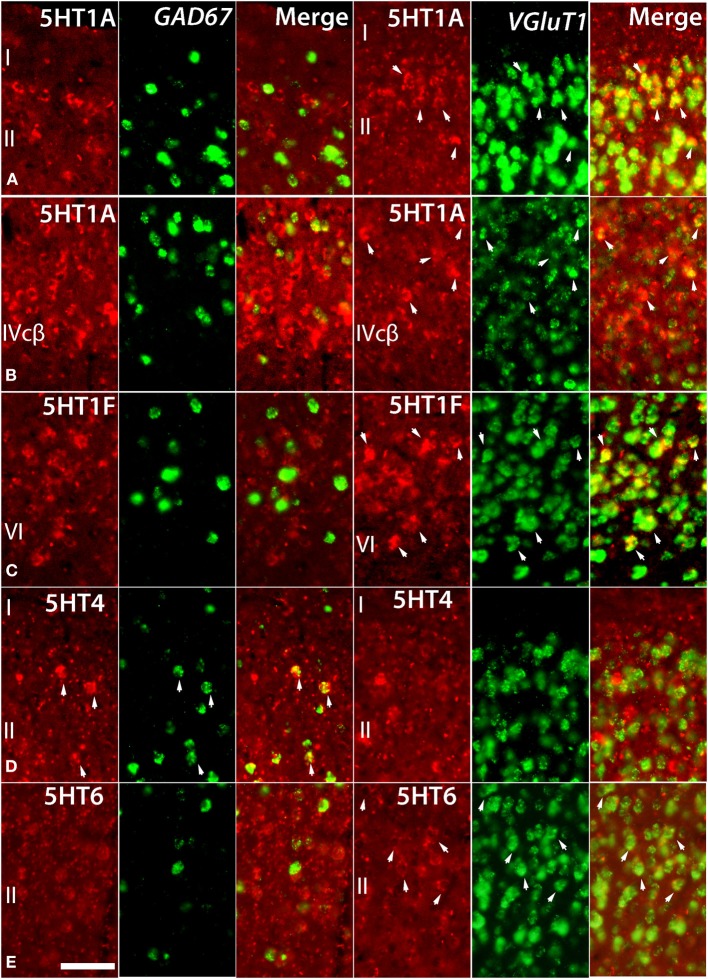
**Double ISH of *5HTR*s (red, DIG) with *GAD67* and *VgluT1* neuronal markers (green, FITC)**. 5HT1A, *5HT1F*, *5HT4*, and *5HT6* with *GAD67* and *VgluT1* neuronal markers in marmoset V1. *5HT1A* in layers II **(A)** and IVcβ **(B)**, *5HT6* in layer II **(E)**, and *5HT1F* in layer VI **(C)** were not expressed in *GAD67*-positive inhibitory cells but were expressed in *VgluT1*-positive excitatory cells. *5HT4* in layer II **(D)** was expressed in *GAD67*-positive inhibitory cells but not in *VgluT1*-positive excitatory cells. The arrow heads indicate the positive signals and coexpressions. Scale bar, 50 μm.

*5HT1B* and *5HT2A* showed high expression levels selectively in V1 and *5HT1A* and *5HT1F* were specifically expressed in V1 (Figure 3). The high expression levels of *5HT1B* and *5HT2A* in V1 were previously reported in macaques (Watakabe et al., 2009), and marmosets (Takahata et al., 2012). In the present study, we found a relatively low level thin band like pattern of expression of *5HT1A* in layer IV Cβ (Figure 3C), which differed from that of macaques and the expression level of *5HT1F* was moderate to high in layer VI (Figure 3E), which was observed to be very low in macaques. When examined by double ISH with excitatory *VgluT1* or inhibitory *GAD67* neuronal marker probes, both *5HT1A* and *5HT1F* were found to be exclusively expressed in excitatory neurons (Figures 4B,C). We also observed that serotonergic projections were dense in layers IV and VI (Figure 3B and Figure S1A), where these four subtypes were expressed. The expressions of *5HT1A*, *5HT1B*, and *5HT2A* overlapped with highly dense serotonergic terminations in layer IV and that of *5HT1F* overlapped with moderately dense terminations in layer VI (Figure 3B). The expressions of the four genes and the serotonin terminations formed sharp boundaries between V1 and V2 (Figures 3A–F).

**Figure 5:**
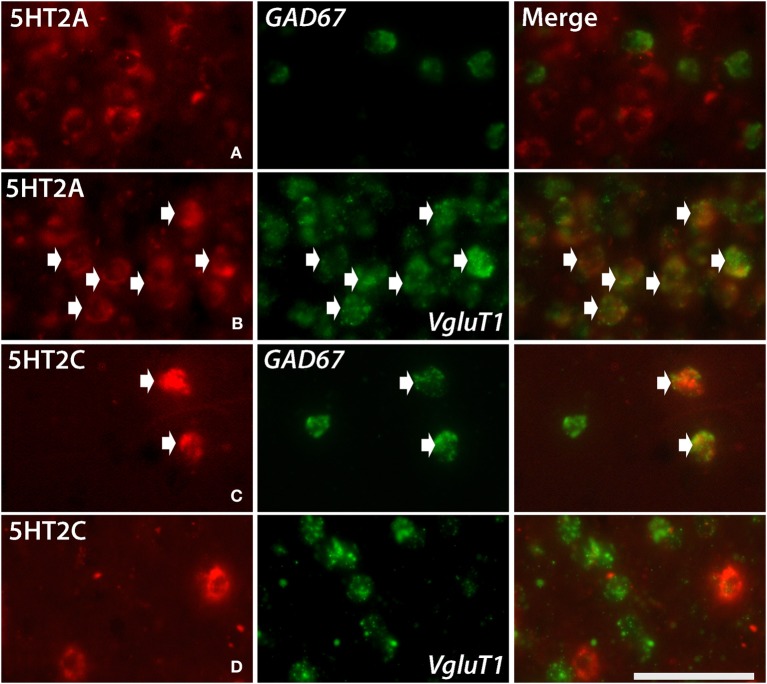
**Double ISH of *5HT2C* and *5HT2A* (red, DIG) with *GAD67* and *VgluT1* neuronal markers (green, FITC)**. *5HT2A* with *GAD67* in layer III of V1 **(A)**, *5HT2A* with *VgluT1* in layer III of V1 **(B)**, *5HT2C* with GAD67 in layer V of frontal cortex **(C)** and *5HT2C* with *VgluT1* in layer V of frontal cortex **(D)**. The arrows indicate the positive signals and coexpressions. Scale bar, 50 μm. Note that the density of *VgluT1* positive excitatory neuron we observed in layer V is less than other layers **(D)**, which is consistent with the result shown in another report (Gittins and Harrison, 2004).

### Serotonin receptor mRNA expressions in hippocampus

**Figure 6:**
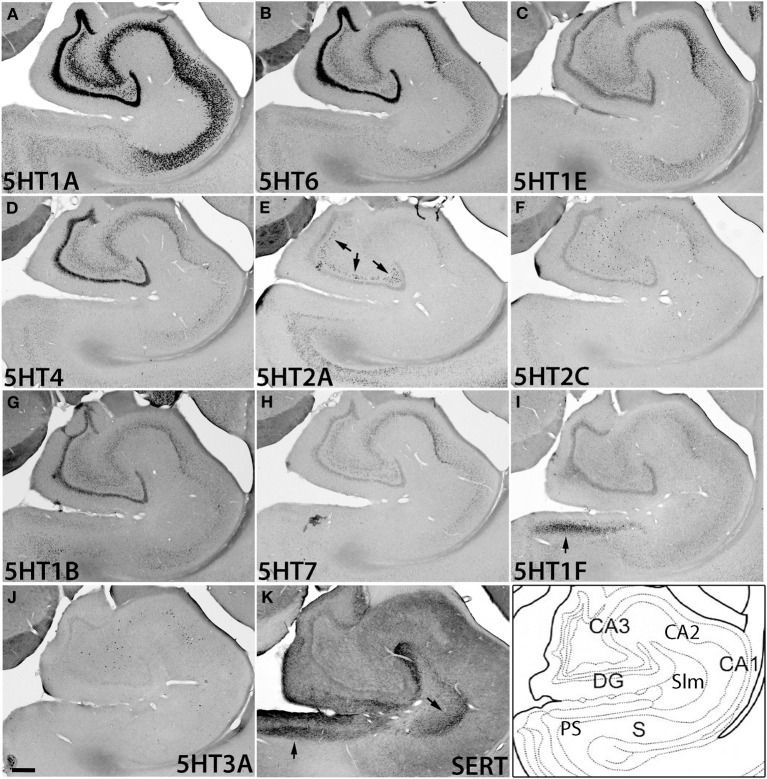
**ISH expression profiles of *5HTR*s in hippocampus**. *5HTR* mRNA expressions **(A**–**J)** and immunohistochemical staining with anti-SERT antibody **(K)** in CA1 and CA3 fields, dentate gyrus (DG), presubiculum (PS), subiculum (S), and stratum lacunosum moleculare (Slm) of hippocampal formation. Arrows for *5HT1F* **(I)**, *5HT2A* **(E)**, and SERT **(K)**, show the corresponding similarity of expressions and innervations in the mouse (see Figures S5C,D,F). Images are adjusted at contrasts that show the clearest image for each *5HTR*. Scale bar, 200 μm.

The hippocampal region consists of the dentate gyrus (DG), CA fields, and subiculum (S) (Figure 6). It was densely innervated by serotonergic terminals in the areas with no receptor expression and stratum lacunosum moleculare (Slm) (Figure 6K). Interestingly, the expressions of *5HTR* mRNAs in the hippocampus were highly subregion-specific. *5HT1A*, *5HT6*, *5HT1E*, and *5HT4* mRNAs, which are expressed in the cortical upper layer, were all abundantly expressed in the DG and pyramidal cell layer from CA3 to CA1. Among them, *5HT1A* mRNA showed particularly prominent expression throughout these structures, whereas the other *5HTR* mRNAs exhibited relatively weak expression in CA3.

In contrast to this group of genes, *5HT2A* and *5HT2C* mRNAs as well as *5HT3A* mRNA exhibited characteristically scattered expressions in the polymorph layer of DG (*5HT2A*) and CA fields (*5HT2C* and *5HT3A*) (Figures 6E,F,J). Note that these three mRNAs showed very low expression levels in granule cells, no higher than the expression level of the sense probe, which showed nonspecific faint background staining in DG. Such scattered expression suggests that they are expressed in inhibitory neurons. Indeed, by double ISH we confirmed that the *5HT2C* and *5HT3A* mRNAs in the hippocampus were expressed in a subset of *GAD67*-positive inhibitory neurons (data not shown). The observation that the expression distribution and density differed among *5HT2A*, *5HT2C*, and *5HT3A* mRNAs (Figure S2) suggests that they are expressed in different types of cell.

Despite dense projection by serotonergic terminals, *5HT1F* was the only subtype expressed in the presubiculum above a moderate level. Other receptor types were distributed sparsely and expressed only at low levels (Figure S2, S).

### Serotonin receptor mRNA expression in thalamus, hypothalamus, and amygdala

Regarding subcortical regions, we examined the thalamus, hypothalamus, amygdala, caudate, septum, ventral striatum, and superior colliculus. Overall, the repertoires of *5HTR* subtypes expressed were quite limited in the thalamus, and as in V1 of the cortex, many regions showed conspicuous overlap between mRNA expression and serotonergic termination as described below.

We examined the expression patterns in a few conspicuous nuclei (as described below) belonging to various groups of the thalamus. Overall, in terms of the number of receptor types expressed, the thalamus showed the least receptor diversity (see Table 3). We did not observe the expressions of *5HT1E*, *5HT1F*, *5HT3A*, and *5HT4* in any subnuclei at levels above the background level. The serotonergic terminations into the thalamus were heterogeneous and showed laterally low and medially high gradations (see Figures S1B,E). Both the medial geniculate nucleus (MG) (Figure S3K) and the lateral geniculate nucleus (LG) (Figure S4K) had moderate and heterogeneous serotonergic terminations.

**Figure 7:**
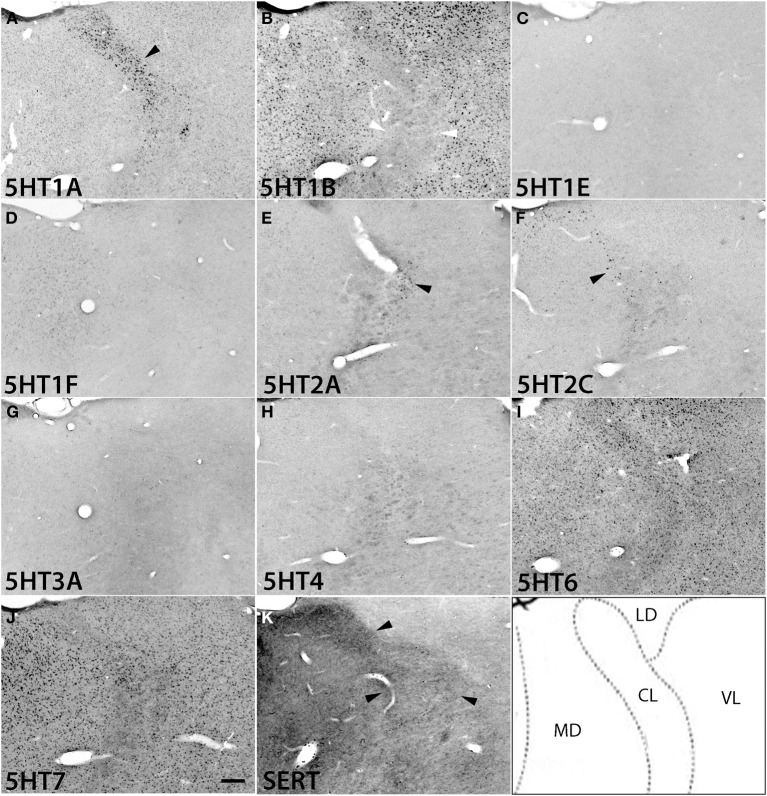
**ISH expression profiles of *5HTR*s in thalamus**. *5HTR* mRNA expressions **(A–J)** and immunohistochemical staining with anti-SERT antibody **(K)** in central lateral (CL), mediodorsal (MD), lateral dorsal (LD), and ventral lateral (VL) thalamic nuclei. The black arrowheads in **(A)**, **(E)**, and **(F)** show the overlap of *5HT1A* **(A)**, *5HT2A* **(E)**, and *5HT2C* **(F)** expressions with corresponding dense serotonergic projections at CL **(K)** (also see Figure S1), whereas the white arrowheads in **(B)** show the corresponding mismatch between *5HT1B* expression and projections at CL **(K)**. Images are adjusted at contrasts that show the clearest image for each *5HTR*. Scale bar, 200 μm.

*5HT1A* showed a high level of mRNA expression in the CL nucleus (Figure 7A), which overlaps with the dense serotonergic termination in CL (Figure 7K, also see Figures S1B,C). In sharp contrast, *5HT1B* showed little expression in CL but was expressed at high levels from nuclei lateral dorsal (LD), ventral lateral (VL), and mediodorsal (MD) cortices to CL, where the *5HT1A* mRNA expression levels were very low to low. *5HT2A* and *5HT2C* were both sparsely expressed in CL and were little expressed from nuclei medial and lateral cortices to CL (Figures 7E,F). *5HT2C* was also expressed near the midline thalamic nuclei where the serotonergic projections were dense (Figure S1D,E). *5HT6* and *5HT7* were expressed in CL, VL, LA, and MD from very low to low and from low to moderately high levels, respectively.

The overall expression patterns of all the *5HTR* subtypes were similar in the posterior nuclei including the medial, lateral, and inferior pulviner (Figure S2), medial geniculate nucleus (Figures S2, S3), and ventral posterior nuclei including the ventral posterior lateral (VPL), and ventral posterior medial (VPM) nuclei (Figures S2, S4). In the lateral geniculate nucleus (LG), *5HT1A* and *5HT6* were expressed at very low levels, *5HT7* at a low level (Figure S2), and *5HT1B* at a high level (Figures S2, S4). Finally, in the reticular nucleus (RT), *5HT1B*, *5HT2A*, and *5HT2C* were expressed at moderately high levels and *5HT1A* from very low to low levels (Figure S2).

**Figure 8:**
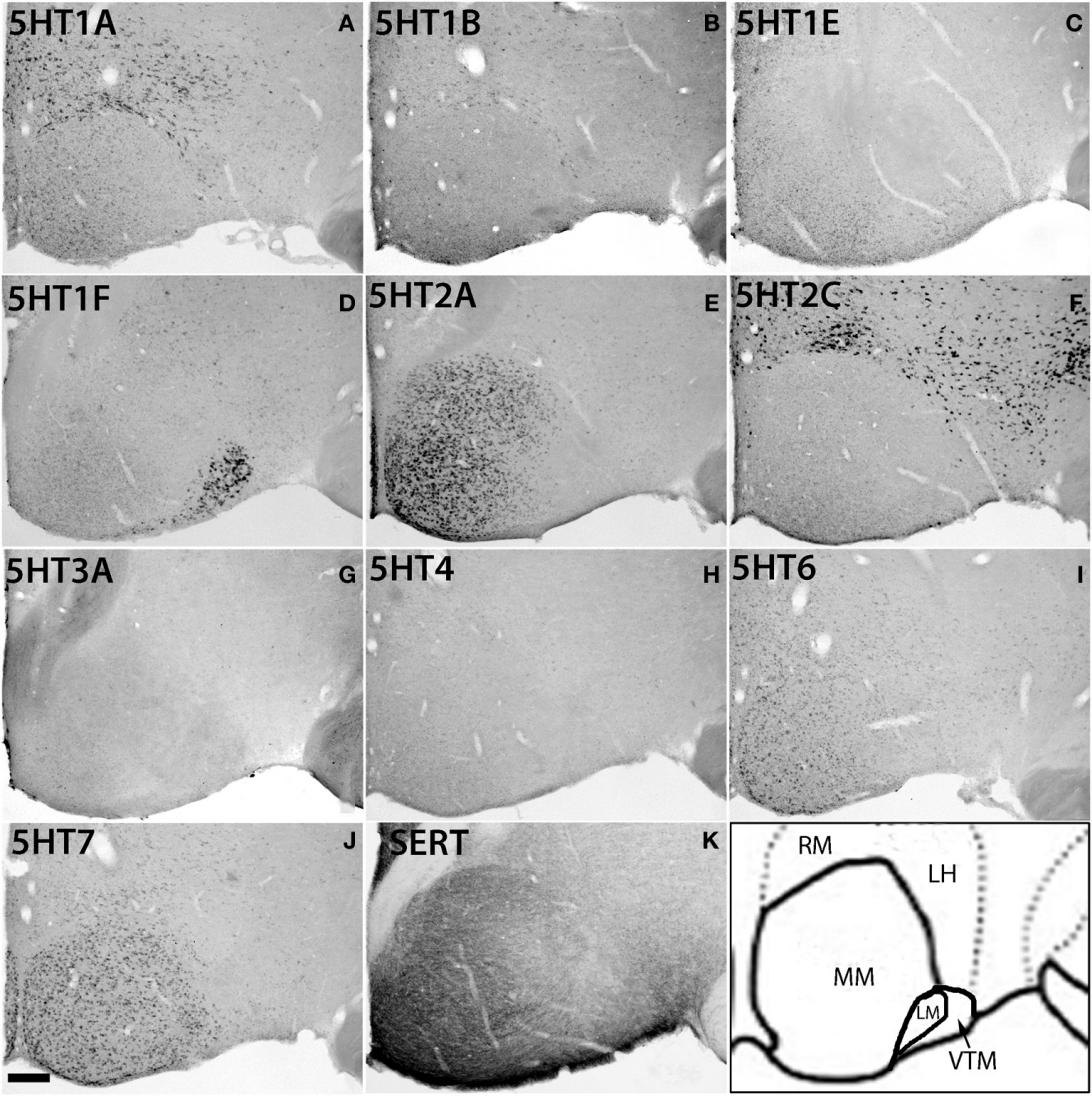
**ISH expression profiles of *5HTR*s in hypothalamus**. *5HTR* mRNA expressions **(A**–**J)** and immunohistochemical staining with anti-SERT antibody **(K)** in lateral (ML), medial (MM), and ventral tuberomammillary (VTM) nuclei of hypothalamus. We observed the striking complementary relationship between the *5HT2A* **(E)** and *5HT2C* **(F)** expressions and overlap of *5HT2A* and *5HT7* expressions with projections at MM. Note that the *5HT1A* **(A)** expression that overlapped with serotonergic innervations in CL (Figure 7A) did not match with the projections at MM. Images are adjusted at contrasts that show the best image for each *5HTR*. Scale bar, 100 μm.

Within the hypothalamic nuclei, the mammillary nucleus exhibited conspicuous heterogeneity of *5HTR* mRNA expressions (Figure 8). Such heterogeneity corresponded to the density of serotonergic projections (Figure 8K). The medial part of the mammillary nucleus (MM) received denser serotonergic projections than the retro-hypothalamus (RH), lateral hypothalamus (LH) and LM nucleus which lie dorsal, lateral and ventro lateral to MM, respectively (Figure 8, reference) The distribution of *5HTR* mRNAs was specific in these regions, which conspicuously overlapped with the serotonergic projections: *5HT2A* and *5HT7* mRNAs were densely expressed in MM but were absent in RH, LH, and LM (Figures 8E,J), and *5HT6* mRNA was also more highly expressed in MM, although it was expressed in both RH and MM. In contrast, we observed a moderately high expression level of *5HT1A* mRNA, very low to low expression levels of *5HT1B* mRNA, and a high expression level of *5HT2C* mRNA in RH, LH, and LM but not in MM. *5HT1E*, *5HT3A*, and *5HT4* mRNAs were expressed at insignificant levels.

**Figure 9:**
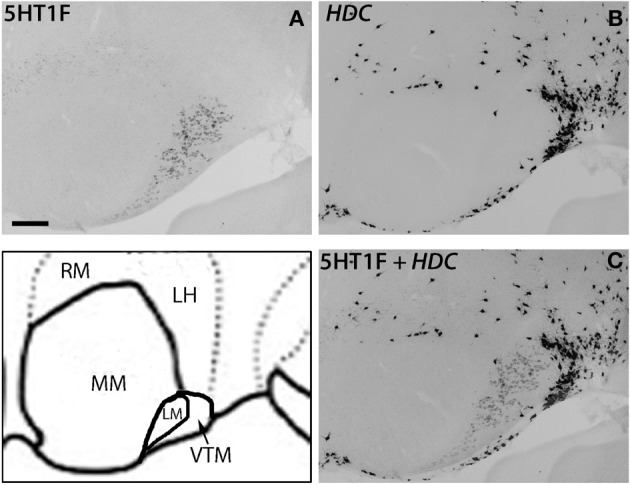
**ISH expression profiles of *5HT1F* and *HDC* in hypothalamus**. *5HT1F* mRNA expression in ML **(A)**, *HDC* mRNA expression in VTM **(B)**, and overlay image of *5HT1F* and HDC mRNA expressions **(C)**. Scale bar, 100 μm.

There was some ambiguity in assigning the localization of *5HT1F* mRNA expression, which was at a high level exclusively in the nucleus lateral to MM, which could be either LM or the ventral tuberomamillary nucleus (VTM) (Figure 8D). VTM, which is part of tuberomamillary nucleus (TM), shows the densest population of histaminergic neurons and can be identified using histidine *HDC* as a marker (Ericson et al., 1987; Sakai et al., 2010). *5HT1F* if present in histaminergic neurons can directly modulate the regulation of these neurons. To examine this possibility and locate *5HT1F* expression, we performed ISH of *5HT1F* and *HDC* in adjacent sections (Figure 9). Our result shows that *5HT1F* and HDC were expressed in a complementary manner, suggesting that *5HT1F* is expressed exclusively in LM.

**Figure 10:**
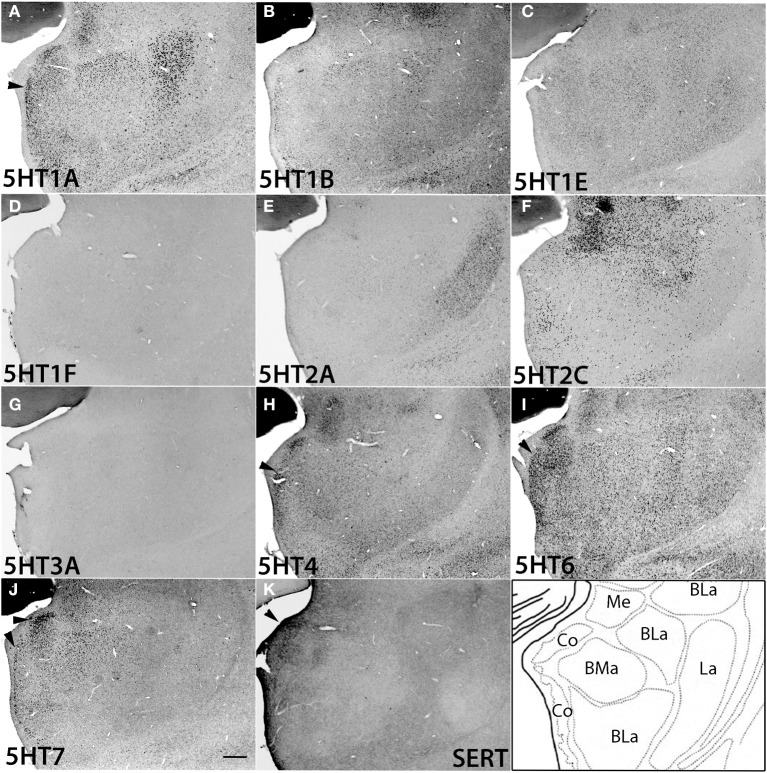
**ISH expression profiles of *5HTR*s in amygdala**. *5HTR* mRNA expressions **(A–J)** and immunohistochemical staining with anti-SERT antibody **(K)** in basomedial (BMa), basolateral (BLa), cortical (Co), lateral (La), and medial (Me) amygdaloid nuclei of amygdala. Note that the arrowheads for *5HT1A* **(A)**, *5HT4* **(H)**, *5HT6* **(I)**, and *5HT7* **(J)** show the overlap of the expressions with serotonergic projections **(K)** near Co. Images are adjusted at contrasts that show the clearest image for each *5HTR*. Scale bar, 200 μm.

The amygdala consists of several subnuclei connected with each other (Figure 10). *5HT1F* and *5HT3A* showed no detectable signals above the background in the amygdala. ISH signals of other *5HTR* subtypes were generally observed in most parts of the amygdala, although signals were heterogeneous and not as pronounced as those in the mammillary nucleus. *5HT1A*, *5HT4*, *5HT6*, and *5HT7* mRNA showed high expression levels in the cortical amygdaloid nucleus (Co), where there were dense serotonergic projections. *5HT1A* mRNA was highly expressed in the basolateral (BLa), basomedial (BMa) and Co and not expressed in the La. *5HT2A* mRNA was expressed only in La and not in Bla, BMa, or Co. *5HT2C* was expressed densely in the medial amygdaloid nucleus (Me), and the expression became very sparse toward La. *5HT1B* mRNA was faintly expressed and *5HT1E* mRNA was homogeneously expressed at low to moderately high levels across all the nuclei. *5HT4* and *5HT7* mRNAs were generally expressed toward the medial part, mostly in Co. The *5HT6* mRNA expression levels were high in Co and low in other nuclei.

### Serotonin receptor mRNA expressions in superior colliculus

**Figure 11:**
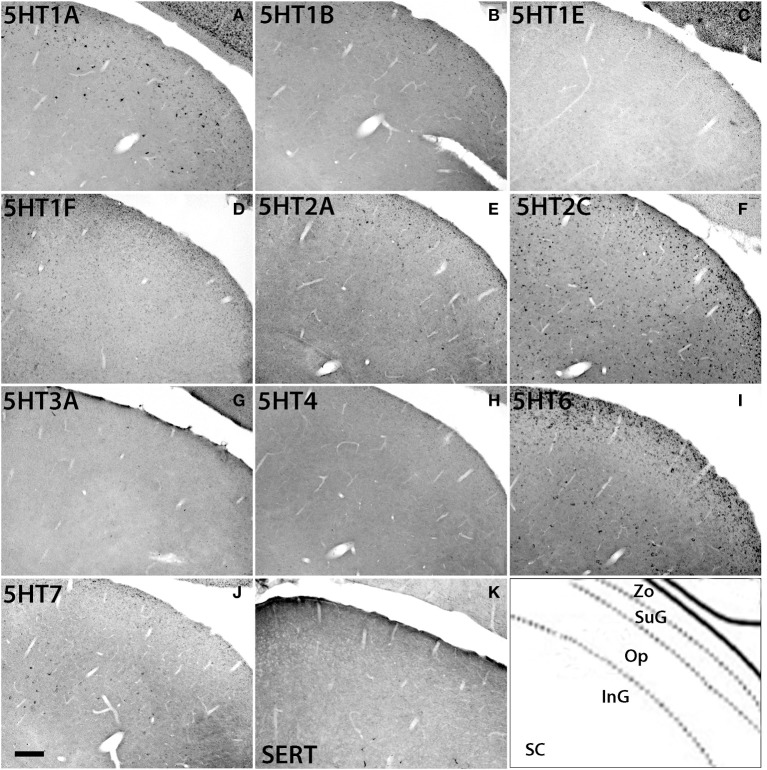
**ISH expression profiles of *5HTR*s in superior colliculus**. *5HTR* mRNA expressions **(A–J)** and immunohistochemical staining with anti-SERT antibody **(K)** in zonal layer (Zo), superficial gray (SuG), optic nerve layer (Op), and intermediate gray (InG) of superior colliculus (SC). Images are adjusted at contrasts that show the clearest image for each *5HTR*. Scale bar, 100 μm.

The *5HTR* subtypes expressed in the superior colliculus (SC) (Figure 11) were similar to those in MD, the adjacent substructure of the thalamus. In SC, we did not find any significant expression of *5HT1F*, *5HT3A*, or *5HT4*. All the other *5HTR* subtypes were sparsely expressed at various levels. The serotonergic projections in SC were moderately dense and appeared to overlap with *5HT6* expression in the zonal layer (Zo). *5HT1A* was mostly expressed in superficial layers including the zonal layer, superficial gray (SuG) layer, and optical nerve layer (Op), and its expression levels ranged from moderately high to high depending on the cell type. *5HT2A* and *5HT1B* were expressed at very low and low levels, respectively, in Zo and SuG. *5HT1E* was exclusively expressed in Zo at a low level. *5HT2C* was expressed across the superior colliculus at a moderately high level; its expression was generally dense in Zo and SuG. *5HT6* was expressed at a moderately high level in two tiers, densely in Zo and SuG, and sparsely in the intermediate gray (InG) layer. Finally, *5HT7* was expressed at a low level in InG.

### Serotonin receptor mRNA expressions in caudate and septum

**Figure 12:**
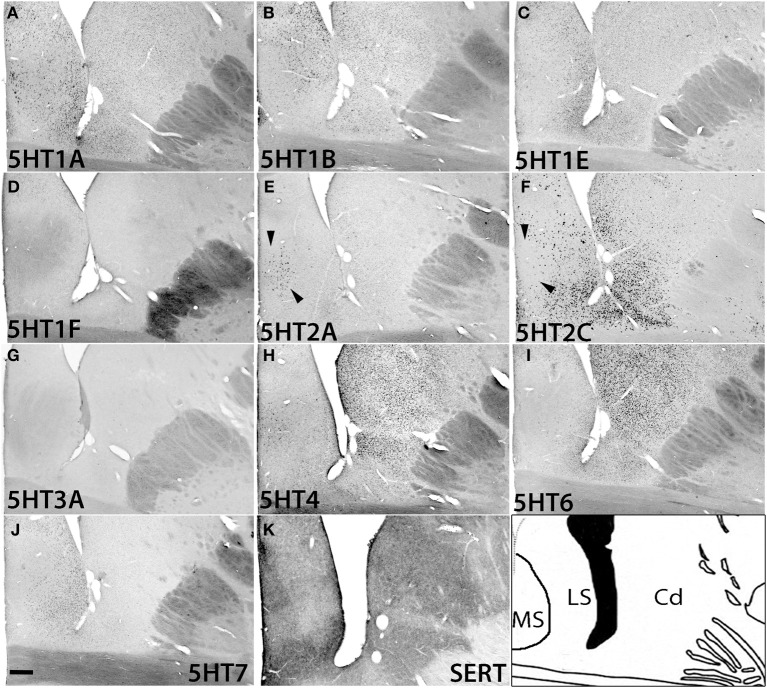
**ISH expression profiles of *5HTR*s in caudate and septum**. *5HTR* mRNA expressions **(A–J)** and immunohistochemical staining with anti-SERT antibody **(K)** in the caudate (Cd) nucleus, and medial septum (MS), and lateral septum (LS). Note that the arrowheads for **(E)** and **(F)** show the presence and absence of *5HT2A* and *5HT2C* expression, respectively, in the medial septum. Images are adjusted at contrasts that show the clearest image for each *5HTR*. Scale bar, 200 μm.

In the caudate, medial septum (MS), and lateral septum (LS) (from right to left in Figure 12), the serotonergic projections varied and showed no apparent overlap with 5HT expression. In the caudate, *5HT1F* and *5HT3A* were not expressed. *5HT1E* and *5HT7* were faintly expressed.The mRNA expression levels were low for *5HT1A*, moderately high for *5HT1B*, and moderately high to high for *5HT6* and *5HT4*. *5HT2C* at moderately high mRNA expression levels was densely expressed toward the medial part (Figure S2) and more scattered toward the lateral part of the caudate (Figure 12F).

In the septum, *5HT1F* and *5HT3A* were not expressed. *5HT1A* showed sparse but significant expression in both the medial septum (MS) and lateral septum (LS). *5HT1B* showed a moderately high mRNA expression level, *5HT1E* and *5HT7* were expressed at low levels, and *5HT6* was faintly expressed in the lateral septum. *5HT4* was generally expressed at moderately high to high levels in the medial septum. *5HT2A* was exclusively expressed in the medial septum at a moderately high level, and complimentarily *5HT2C* was expressed at a moderately high level in the lateral septum (indicated by arrow heads in Figures 12E,F)

### Serotonin receptor mRNA expressions in ventral striatum

We examined the 5HT expression patterns in the internal globus pallidus (iGP), and external globus pallidus (eGP), substantia nigra pars reticulate (SNr), and substantia nigra pars compacta (SNc), representing the ventral striatum. The serotonergic projections in these regions were again heterogeneous. In SNc, the projection density increased near the inferior regions where the expression was generally denser. In the globus pallidus (Figure S2), a small repository of 5HT subtypes was expressed and we did not detect signals above the background level for *5HT1B*, *5HT1F*, *5HT4*, *5HT3A*, or *5HT7* in both nuclei. All the 5HT subtypes were sparsely expressed in these nuclei. The mRNA expression levels were very low for *5HT1A* and low for *5HT1E*, *5HT2A*, and *5HT6* in both the iGP and eGP. Interestingly, *5HT2C* was expressed in the iGP and eGP at high and very low levels, respectively (Figure S2).

**Figure 13:**
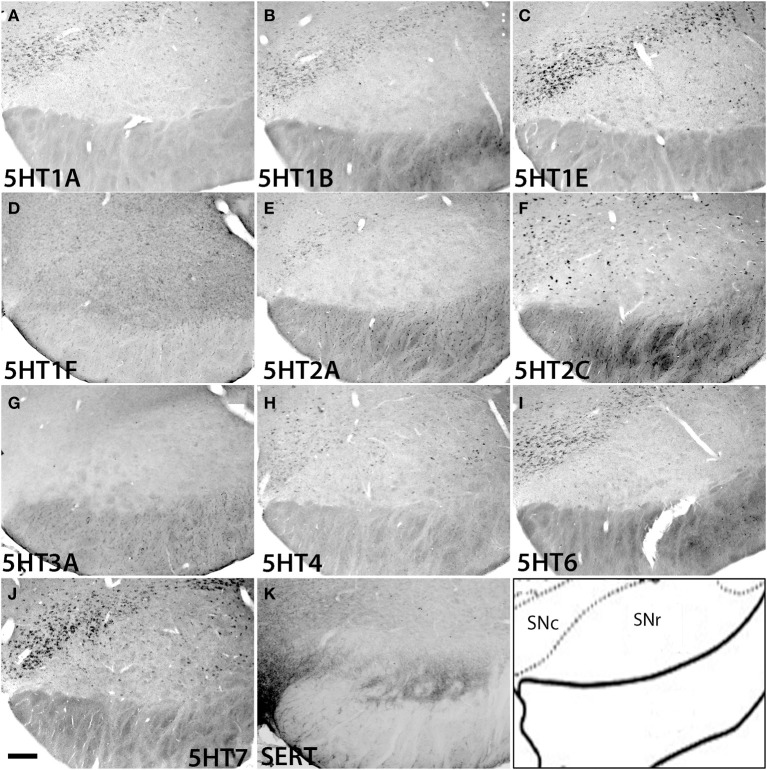
**ISH expression profiles of *5HTR*s in substantia nigra**. *5HTR* mRNA expressions **(A–J)** and immunohistochemical staining with anti-SERT antibody **(K)** in pars compact (SNc) and pars reticular (SNr) of substantia nigra. Images are adjusted at contrasts that show the clearest image for each *5HTR*. Scale bar, 100 μm.

In the substantia nigra (Figure 13 and Figure S2), *5HT1F* and *5HT3A* were not expressed. In SNc, *5HT2C* and *5HT4* mRNAs were expressed sparsely whereas mRNAs of other 5HTs were expressed densely. The levels of expression were very low for *5HT2A*, low for *5HT1A* and *5HT1B* and moderately high for *5HT1E*, *5HT2C*, *5HT4*, *5HT6*, and *5HT7*. In SNr all the 5HTs were expressed sparsely at very low levels except *5HT2C*, which was expressed sparsely but at a high level (Figure 13).

## Discussion

We report the mRNA localization of all the 10 *5HTR*s that are expressed, as well as the distribution of serotonin terminations in the marmoset brain. Besides confirming the published results of numerous previous studies, the present study notably demonstrates several new findings about the organization of serotonergic systems. On the basis of our findings we discuss the possible roles of *5HTR*s in the marmoset brain, as revealed by our analysis of overall expression patterns.

### Technical considerations

In our present study we were unable to obtain the results for *5HT1D*, *5HT3B*, and *5HT5A*. When checked for their expression patterns in the human data set (ABA, 2012), we were unable to find the expression of 5HT1D and *5HT3B*, suggesting that the absence of expression found in our study is not due to artifact. *5HT5A* is found in the frontal cortex at low levels in both humans (ABA, 2012) and mice (Goodfellow et al., 2012, Figure S6). On the basis of this finding, we could not exclude the possibility that ISH using our *5HT5A* probes might have failed to detect low signals. We also encountered some constant background signals associated with the expression of *5HT1F* and *5HT1E*, and we were unable to detect signals for *5HT1E* when testing for its presence using excitatory or inhibitory, neuronal markers for double hybridization. On the basis of our previous study (Watakabe et al., 2007) we consider that low mRNA expression levels of *5HT1F* and *5HT1E* might be the reason for the granular background and also both the lower mRNA expression level and high GC content of *5HT5A* (63.41%) than of the other *5HTR*s might be the reason for the failure to detect ISH signals.

### Overlap of serotonin receptor mRNA distribution and serotonergic terminations

Serotonergic projections in the marmoset brain were generally associated with serotonin receptor expressions. Our data show a marked overlap of the mRNA expressions of most *5HTR*s with serotonergic terminations in the visual cortex (Figure 3), the subiculum (Figure 6I), the CL nucleus of the thalamus (Figure 7A,E,F, also see Figures S1B–E), the medial mammillary nucleus (Figures 8E,J), the cortico amygdaloid nucleus of the amygdala (Figure 10), and the midline thalamic nuclei (Figure S1). All the subtypes, except *5HT1B*, that showed overlaps have somatodendritic localization of their receptor proteins (Table S2), suggesting a strong correlation between serotonin availability and receptor expression.

Interestingly, none of the *5HTR*s were expressed in layer I where corresponding serotonergic termination were present and were relatively high in density at certain areas (Figure 3). Likewise, both in the mouse and marmoset no serotonergic terminations were found in the pyramidal layer of the hippocampus, where all the *5HTR*s are expressed; instead they were more prominent in Slm (Figure 6 and Figure S5). Both layer I of cortex (Shipp, 2007) and Slm (Maccaferri, 2011) of the hippocampus receive the apical tuft of pyramidal cell dendrites. This mismatch suggests that the major target of serotonergic terminations in the supragranular layer of the cortex and hippocampus is the apical dendritic tuft of neurons, which is known to increase the gain of pyramidal neurons (Larkum et al., 2004).

### Cortical expressions of 5HTRs and circuitry implications

In summary, the upper (supragranular), middle, and lower (infragranular) layers showed quite different patterns of *5HTR* expressions. This feature of *5HTR*s having different mRNA expression patterns in different layers suggests distinct roles of *5HTR*s in the primate cortex that presumably affect the function of each layer.

Large varicose serotonergic fibers originating from the median raphe nucleus (MRN) have been reported to project at the supragranular layers in the marmoset (Hornung et al., 1990) and macaque (Wilson and Molliver, 1991). These innervations form synapses with supragranular inhibitory neurons in a basket like pattern in macaques and chimpanzees but not in humans (Raghanti et al., 2008), and in both cats and marmosets such a basket like pattern is observed in calbindin-positive (CB+) interneurons (Hornung and Celio, 1992). In the rat hippocampus also innervation to CB+ inhibitory neurons has been reported (Freund et al., 1990). The interneurons are likely to inhibit the nearby pyramidal cells; as has been demonstrated in many locations of the cortex (Sheldon and Aghajanian, 1990; Ropert and Guy, 1991; Foehring et al., 2002).

We report expression of *5HT4* mRNA in *GAD67*-positive inhibitory neurons and the expressions of *5HT1A* and *5HT6* mainly in *VgluT1*-positive excitatory neurons in the upper layers of V1 (Figure 4). Thus, *5HT4*, which has excitatory cellular effects (Table S2), might indirectly inhibit neighboring pyramidal neurons and *5HT1A*, which has an inhibitory cellular effect, might be recruited to directly inhibit pyramidal neurons. *5HT6*, which has an excitatory cellular effect, similarly can be supposed to excite pyramidal neurons.

Direct and indirect inhibition might be recruited separately, depending on the two different populations of terminal axons originating from different raphe nuclei with their unique behavioral consequences. MRN forms a direct synaptic contact with neuronal somata, whereas DRN has a widespread effect through volume or extrasynaptic transmission (Törk, 1990; Michelsen et al., 2007). The MRN innervation forms synaptic contact with CB+ interneurons (as mentioned above), which on the basis of our findings seem to express *5HT4*. Interestingly, *5HT4* has also been detected in certain CB+ enteric neurons of rodents (Poole et al., 2006). Our observation of *5HT1A* expression mainly in excitatory neurons is based on visual inspection in V1, but previous reports have shown that in Layer II of the monkey prefrontal cortex (PFC) 83% of *5HT1A* is expressed in *VgluT1* positive excitatory neurons and 43% of the remaining inhibitory neurons are found in CB+ interneurons. This suggests that *5HT1A* may be recruited by both MRN and DRN in PFC.

The extrasynaptic localization of *5HT1A* receptors (Riad et al., 2000) supports the idea of direct inhibition of pyramidal neurons expressing *5HT1A* (Figure 4) by volume transmission triggered by DRN. In summary, *5HT4* might be recruited in synaptic-indirect inhibition of pyramidal neurons by the stimuli originating from MRN whereas *5HT1A* might be recruited in extrasynaptic-direct inhibition of pyramidal neurons by the stimuli originating from DRN.

### Thalamic nuclei projecting to the cortex show less receptor diversity

In thalamic nuclei projecting to cortex, only *5HT1A*, *5HT1B*, *5HT6*, and *5HT7* were prominently expressed. *5HT1A* and *5HT1B* have inhibitory cellular effects (Table S2) whereas *5HT6* and *5HT7* have excitatory cellular effects (Table S2). This suggests that the cortically projecting thalamic nuclei, maintain a balance between excitatory and inhibitory effects on inputs and outputs only by recruiting a limited subgroup of *5HTR*s. *5HT2C*, and *5HT2A* were expressed in addition to these four *5HTR* subtypes in the CL, which projects to the striatum(Van der Werf et al., 2002), and in the RT, which receives inputs from the cortex (Smith, 2008). Taken together, our data suggest that those regions of the thalamus, which gates afferent information to the cortex, have fewer *5HTR* subtypes (see Table 3 and Figure S2) and in contrast, the cortex, which integrates sensory information, has more *5HTR* subtypes. Aligning to our findings, physiological data collected from the ferret thalamus (Monckton and McCormick, 2002) also suggest that serotonin has lesser influence (direct postsynaptic inhibitory) on the primary sensory nuclei than on the intralaminar nuclei.

### Complementary expression of 5HT2A and 5HT2C

Many studies have suggested independent, reciprocal, opposing and balancing functional features associated with *5HT2A* and *5HT2C* receptors (Popova and Amstislavskaya, 2002; Winstanley et al., 2004; Nonogaki et al., 2006; Aloyo et al., 2009; Halberstadt et al., 2009). In the hypothalamo-pituitary-testicular -based system, the neural control of male sexual motivation and arousal involves the facilitative action of *5HT2A* and suppressive action of *5HT2C* in a reciprocal manner (Popova and Amstislavskaya, 2002). In the hypothalamus of obese A^y^ mice, *5HT2A* and *5HT2C* receptors are suggested to have reciprocal roles in the regulation of feeding and energy homeostasis (Nonogaki et al., 2006). The complementary expression of *5HT2A* and *5HT2C* observed in the hypothalamus in our study (Figures 8E,F) is consistent with the finding of Papova et al. and Nonogaki et al. in nonprimates. Besides the hypothalamus, the septum (Figures 12E,F) and entorhinal cortex (Figure 2, k5,k6) also showed complementarity. In V1, there was an enriched expression of *5HT2A* in contrast to the scant expression of *5HT2C* (Figure 2).

*5HT2A* is expressed in 86 to 100% of upper layer glutamatergic cells and in 13–31% of inhibitory cells in the monkey and human PFC (De Almeida and Mengod, 2007). Similarly, in the marmoset and macaque V1, it is also mostly expressed in the excitatory neurons (Watakabe et al., 2009; Nakagami et al., 2013, Figure 5). In contrast, the expression of *5HT2C* was scant and was mostly detected in the inhibitory neurons (Figure 5) of layer V. In rats, *5HT2C* is primarily expressed in excitatory neurons in the PFC (Puig et al., 2010). This difference may be species-specific between the marmoset and rat or due to the difference in the equivalent ages of the two animal species used. In rats there is high expression of *5HT2C* in layers IV and V until P14, and after P56, the expression level becomes low and is limited to layer V (Li et al., 2004; Jang et al., 2012). Overall, our data supports the functional complementarity between *5HT2A* and *5HT2C* suggested in previous pharmacological studies.

### Sporadic and highly localized expressions of 5HT1F and 5HT3A

*5HT1F* is only expressed in layer VI of V1 (Figure 3), the presubiculum (Figure 6), and LM of the hypothalamus (Figure 9). In V1 and the presubiculum, its expression overlapped with dense serotonergic terminations, again suggesting a high turnover rate of serotonin at these sites. In mouse V1, a recent study has shown that layer VI works as a major mediator of cortical gain modulation (Olsen et al., 2012). Our previous work shows the role of *5HT1B* in increasing the signal-to-noise ratio and *5HT2A* in gain control in V1 (Watakabe et al., 2009). In this report, we have shown the expression of *5HT1F* in excitatory neurons of layer VI. Together, these findings suggest for possible recruitment of the *5HT1F* receptor present in layer VI for supporting the visual gain function in marmoset.

The mammillary body, which includes MM and LM (Vann, 2010) (Figure 8), appears to lack interneurons in primates (Veazey et al., 1982), whereas the TM, which surrounds the mammillary body, is composed of inhibitory neurons only. Surprisingly, the members of the *5HT1* family, which have inhibitory cellular effects (Table S2), are not expressed in the mammillary body, except *5HT1F*. This suggests that serotonin primarily functions to facilitate the excitation of the mammillary body in MM, as revealed by the dense serotonergic innervations and expression of *5HT2A*, *5HT6*, and *5HT7* receptors with excitatory cellular effects (Table S2) but hyperpolarizes the ML by recruiting *5HT1F*, thus balancing the overall excitation of the mammillary body. Overall, the sporadic regional localization of *5HT1F* receptors in the marmoset brain may be related to the mediation of the gain modulation or balancing functions.

The expression profile of *5HT3A* we obtained in the cortex was different from that observed in mice, where it was associated with cortical interneurons. *5HT3A* accounts for nearly 30% of all interneurons and is suggested to be involved in shaping the cortical circuit in rodents (Rudy et al., 2011). In addition, Jakab and Goldman-Rakic (2000) showed the *5HT3A* receptor at the cell body of cortical neurons in macaques. There may be species differences in the expression pattern of *5HT3A* in the cortex between marmosets and other species. In our present study, we examined *5HT3A* expression using several probes of *5HT3A*, but except for the probes mentioned in the results (shown in Table 1) we observed high background signal intensities for all probes. The working probe was found to be expressed only in GABAergic interneurons in the CA fields of the hippocampus (Figure 6J). Therefore, we cannot exclude the possibility that the differences observed in our marmoset study are due to the different isoforms generated by alternate splicing, because two splice variants of *5HT3A* are found in humans, which exhibit similar pharmacological and electrophysiological profiles when expressed as homomers (Hannon and Hoyer, 2008)

### Comparison of 5HTR mRNA expression between different species

*5HT1A* was expressed in the marmoset, but not in the macaque, in layer IV of V1. The expression is also lacking in human V1 (ABA, 2012). It is tempting to correlate this difference with species-specific physiological differences, such as dichromatic vision, observed in some marmosets (Solomon, 2002; Surridge et al., 2003), compared with the trichromatic vision in humans and macaques (Surridge et al., 2003). Besides this difference, features such as the expression of *5HT1A* and *5HT6* in the upper layer, the V1-specific expression of *5HT1B*, the enriched expression of *5HT2A* in V1, the rostral decrease in the expression of *5HT2C*, the low expression level of *5HT7* and the absence of expression of *5HT3A* (as discussed above) in the cortex were very much similar to those in humans (ABA, 2012). Besides these similarities, the upper layer expression of *5HT1A*, which has been observed in the marmoset (in the present study), macaque and human (De Almeida and Mengod, 2008) is also observed in the rat PFC (Goodfellow et al., 2009), and the expression of *5HT7* mRNA, which is observed prominently in the thalamus and at low levels in the cortex, is also similarly observed in rodents (Gustafson et al., 1996). Together, the expressions of *5HT1A* and *5HT7* receptor subtypes in the cortex seem to be conserved between rodents and primates.

In the hippocampus there was a surprising similarity in the expression patterns observed between marmosets and mice. In both species, except for *5HT2C* and *5HT3A*, the expression of all the *5HTR*s was limited only to the pyramidal layer (Figure 6 and Figure S5), suggesting that majority of serotonin receptors are recruited for the modulation of glutamatergic transmission in the hippocampus. The serotonergic projections, in both the species (as discussed above) were dense at Slm (Figure 6 and Figure S5K). The overlap between serotonergic terminations and *5HT1F* observed in the presubiculum, the specific expression of *5HT2A* in the polymorph layer of DG, and high overall expression level of *5HT1A* observed in the marmoset study was very similar to that in mice (Figure 6 and Figure S5). In the thalamus, again the number of receptor subtypes expressed was smaller than that in the cortex (ABA, 2009).

Besides the conspicuous differences in the overall mRNA expression levels of 5HTs (Figure S7), which were low in mice, there are some notable differences between the mouse and marmoset expression profiles observed in the cortex. *5HT1E* found in the marmosets (Figure 1) was not detected in the mice (ABA, 2009), and the enriched and specific expressions of *5HT1A*, *5HT1B*, *5HT1F*, and *5HT2A* found in V1 of the marmosets (Figure 3) were also not observed in the mice (Figure S6). *5HT4* observed in inhibitory neurons of the marmosets was scarcely expressed in the mouse cortex (Figure S6). *5HT3A* is expressed in cerebral cortex of macaques (Jakab and Goldman-Rakic, 2000) but was not observed in our study of the marmosets. In mice it was expressed mainly in upper layers including layer I (Figure S6), where there was no expression of any *5HTR*s in the marmoset. Among the other expression patterns that were exclusively observed in the mice are as follows: the expression of 5HT1D in layer 6b of SS (Figure S6, c2), the sparse expression of *5HT1B* in layer 4 of SS (Figure S6, b2), abundant expression of *5HT1F* in MO (Figure S6, d3).

Taken together, the mRNA expression pattern of *5HTR*s in the marmoset as compared with those in the mouse shows some significant differences in the cortex, which suggests certain primate specific roles of *5HTR*s and the usefulness of the marmoset as a primate model in further studies of serotonergic modulations in higher brain functions that are specific to primates

### Conflict of interest statement

The authors declare that the research was conducted in the absence of any commercial or financial relationships that could be construed as a potential conflict of interest.
