# Supplementary material for: mRNA expression profile of serotonin receptor subtypes and distribution of serotonergic terminations in marmoset brain
Source: Front Neural Circuits. 2014 May 19;8:52. doi: 10.3389/fncir.2014.00052 (PMC4032978; doi:10.3389/fncir.2014.00052)
Supplement: Supplementary file 1 [file Presentation1.PDF]

Supplementary Material

mRNA expression profile of serotonin receptor subtypes and distribution of serotonergic terminations in marmoset brain

Rammohan Shukla[1,2], Akiya Watakabe[1,2] and Tetsuo Yamamori[1,2,*]

[1]Division of Brain Biology, National Institute for Basic Biology, Okazaki, Aichi 444-8585, Japan [2]Department of Basic Biology, Graduate University for Advanced Studies (SOKENDAI), Okazaki, Aichi 444-8585, Japan

***Correspondence:** Dr. Tetsuo Yamamori, National Institute for Basic Biology, Division of Brain Biology, 38 Nishigonaka, Myodaiji, Okazaki, Aichi, 444-8585, Japan.
yamamori@nibb.ac.jp

Supplementary Figure legends

Supplementary Figure S1. (A) SERT immunohistochemistry of V1. Note the dense serotonergic projections in layer IV indicated by black arrowheads. **(B)** More anterior section of thalamus showing characteristic projections at CL and ventricles (black arrow head). C, D, and E show that the expression and serotonergic projections overlap near the ventricle region (black arrow head). Abbreviations are the same as those in Figure 7 and the main text. Scale bars: (A), 100 μm (B), 200 μm; (C, D, and E), 200 μm.

Supplementary Figure S2. Higher-magnification images of different regions described in the text. The boxed images represent the positive signals with different levels of expression, as mentioned in Table 3. Note that all images of a given gene are grouped together and then adjusted to the same level of contrast. Scale bar, 50 μm.

Supplementary Figure S3. ISH expression profiles of *5HTR*s in medial geniculate nucleus (MG). *5HTR* mRNA expressions **(A-J)** and immunohistochemical staining with anti-SERT antibody **(K)** in MG of thalamic area. Images are adjusted at contrasts that show the clearest image for each *5HTR*s. Scale bar, 100 μm.

Supplementary Figure S4. ISH expression profiles of *5HTR*s in thalamic areas. *5HTR* mRNA expressions **(A-J)** and immunohistochemical staining with anti-SERT antibody **(K)** in lateral geniculate nucleus (LG), ventral posteromedial nucleus (VPM), and ventral posterolateral (VPL) nucleus of thalamus. Note that only *5HT1B* (B) shows a conspicuous expression at moderately high levels in LG. Images are adjusted at contrasts that show the clearest image for each *5HTR*. Scale bar, 200 µm.

Supplementary Figure S5. ISH expression profiles of *5HTR*s in mouse hippocampus. *5HTR* mRNA expressions **(A-J)** and immunohistochemical staining with anti-SERT antibody **(K)** in CA1 and CA3 fields, dentate gyrus (DG), presubiculum (PS), subiculum (S) and stratum lacunosum moleculare (Slm) of hippocampal formation. Arrows for 5HT1F **(I)**, 5HT2A **(E)**, and SERT **(K)** show the corresponding similarities for expression and innervations in the marmoset (see Figure 6). Images are adjusted at contrasts that show the clearest image for each *5HTR*. Scale bar, 200 µm.

Supplementary Figure S6. **ISH expression profiles of *5HTR*s in mouse cortex.** ISH expression profiles of *5HTR*s in as visual (VIS), somatosensory (SS) and somatomotor (MO). Layers identified by Nissl staining (not shown) are indicated on the left. Arrows for *5HT1D* highlights the expression in layer 6b of SS. Note that all images of a given gene are grouped together and presented at the same contrast level. Scale bar, 100 µm.

Supplementary Figure S7a, b and c. Laminar profiles of ISH signals quantified by measuring the optical density. (a) and **(b),** Optical density of expression in marmoset cortex corresponding to Figure 1 and Figure 2, respectively, of main text. (c), Optical density of expression in mouse cortex corresponding to supplementary Figure S6. The numeric figure 0, 100 and 200 correspond to pixel values. As all images of a given gene are grouped together and presented at the same contrast level, the comparison is best for a given gene in different areas.

Notes for Supplementary Figure S8a, b, c and d. An example showing the effect of contrast on noise from white matter and the difference between amounts of light emitted by different sections belonging to the same region has been demonstrated. Both raw (color) and processed (contrasted and gray scale) images used for Supplementary Figure S4 (F and J) have been used in the example.

To show the weak signals (S8a and b, white box), the image was given contrast, which also enhanced the noise from the adjacent white matter (S8a and b, black box). Figure S8a (and c) demonstrates the different amount of light emitted by white matter in two sections that belong to the same region (LGN). The black boxes in figures S8c and S8d also show the effect of contrast in enhancing the noise from white matter

Supplementary Table S1. Summary of ISH probes for 11 serotonin receptors in mice. The hybridization temperature for all the *5HTR*s 60°C. The amplicon includes the primer sequence. **F** indicates forward and **R** indicates reverse**.**

Supplementary Table S2. G-protein involved, signaling pathways, postsynaptic potential, and species specific cellular and regional localization for each *5HTR*. ↓ and ↑ represent decrease and increase, respectively. NA: Not available. Note that the details of *5HT1A* is for heteroreceptors only.

Supplementary Table S1

Gene	Primer	Ampilcon Size	GC%	NCBI Accession
5HT1A	**F:** GCTACCAAGTGATCACCTCTCT<br>**R:** TGCACCTCGATCACCTCCAGGG	787bp	60.48	NM_008308
5HT1B	**F:** GGCTACATTTACCAGGACTCCA<br>**R:** TTGGTTCACGTACACAGGAGAC	759bp	57.81	NM_010482
5HT1D	**F:** TCACAGTTGTGAAGCCAAAGGA<br>**R:** TGATAAGCTGTGCCGTGGTGAA	830bp	56.02	NM_008309
5HT1F	**F:** ACAGTTGAGCCTGCCACACCAC<br>**R:** AGTCCGTTGATGGATCGGACAA	837bp	45.40	NM_008310
5HT2A	**F:** GCTGCAGAATGCCACCAACTAT<br>**R:** AGTGTTCACTAAAATTAACTGC	928bp	49.89	NM_172812
5HT2C	**F:** CGTAATCCTATTGAGCATAGCC<br>**R:** CTCCCTCCCAGACAAAGCAGTG	762bp	46.33	NM_008312
5HT3A	**F:** AGTACTGGACTGATGAGTTTC<br>**R:** CAGAGCCATGCACACCACAAA	683bp	51.47	NM_013561
5HT4	**F:** AGAAGGTCGTGCTGCTCACGTT<br>**R:** GGACAGTGTAGTCTATGAAAGG	816bp	51.10	NM_008313
5HT5	**F:** TGCTGGTGCTGGCTACCATCCT<br>**R:** ATGAGGATGCCCACCATGAGGG	700bp	58.00	NM_008314
5HT6	**F:** GCATGAACTGGGCAAAGCTCGA<br>**R:** GAACCAAGTGGATGCTGCCGTA	813bp	62.24	NM_021358
5HT7	**F:** GGCAGAATGGGAAATGTATGGC<br>**R:** GAGAGCTTCCGGTTGATATTCC	655bp	52.06	NM_008315

Supplementary Table S2

Receptor	Major G-Protein, Reference	Signal, Potential (I/E), Reference	Cellular localization : Region Species, Reference
5HT1A	G_i/G_o [1]	↓cellular levels of cAMP, **I**, [2]	**Somatodendritic:** hippocampus, cortex, and others Rat, Monkey [3,4]
5HT1B	G_i/G_o [1]	↓ cellular levels of cAMP, **I**, [2]	**Preterminal Axon:** globus pallidus and substantia nigra Rat; suprachiasmatic Nucleus Mouse [10]
5HT1E	G_i/G_o [1]	↓ cellular levels of cAMP, **I**, [2]	NA
5HT1F	G_i/G_o [1]	↓ cellular levels of cAMP, **I**, [2]	**Somatodendritic:** clustrum, thalamus, amygdala, cortex Guinea Pig [6]
5HT2A	G_q/G_{11} [1]	↑ IP_3 and cytosolic $[Ca^{2+}]$, **E**, [2]	**Somatodendritic:** cortex, hippocampus, septum, basal ganglia, amygdala Rat [4,7] and **Axonal**: cortex Monkey [8]
5HT2C	G_q/G_{11} [1]	↑IP_3 and cytosolic $[Ca^{2+}]$, **E**, [2]	**Somatodendritic:** cortex, amygdala, hippocampus, thalamus Rat,Human [9]
5HT3A	ligand-gated channel	depolarizing membrane, **E**	**Somatodendritic** and **Axonal:** cortex, hippocampus, amygdala Rat [9]
5HT4	G_s [1]	↑ cellular levels of cAMP, **E**, [2]	**Somatodendritic** and **Axonal:** basal ganglia and hippocampus Rat [9]
5HT6	G_s [1]	↑ cellular levels of cAMP, **E**, [2]	**Somatodendritic:** cortex, striatum, hippocampus, and others Rat [5,9]
5HT7	G_s [1]	↑ cellular levels of cAMP, **E**, [2]	**Somatodendritic** and **Axonal:** suprachiasmatic nucleus Mouse [9,10]

References

1. Millan MJ, Marin P, Bockaert J, Mannoury la Cour C (2008) Signaling at G-protein-coupled serotonin receptors: recent advances and future research directions. Trends Pharmacol Sci 29: 454–464. doi:10.1016/j.tips.2008.06.007.

2. Hannon J, Hoyer D (2008) Molecular biology of 5-HT receptors. Behav Brain Res 195: 198–213. Available: http://www.sciencedirect.com/science/article/pii/S0166432808001526. Accessed 28 January 2014.

3. Pompeiano M, Palacios JM, Mengod G (1992) Distribution and cellular localization of mRNA coding for 5-HT1A receptor in the rat brain: correlation with receptor binding. J Neurosci 12: 440–453.

4. Mengod G, Vilaró MT, Raurich A, López-Giménez JF, Cortés R, et al. (1996) 5-HT receptors in mammalian brain: receptor autoradiography and in situ hybridization studies of new ligands and newly identified receptors. Histochem J 28: 747–758. doi:10.1007/BF02272148.

5. Gérard C, Martres MP, Lefèvre K, Miquel MC, Vergé D, et al. (1997) Immuno-localization of serotonin 5-HT6 receptor-like material in the rat central nervous system. Brain Res 746: 207–219. doi:10.1016/S0006-8993(96)01224-3.

6. Riad M, Garcia S, Watkins KC, Jodoin N, Doucet E, et al. (2000) Somatodendritic localization of 5-HT1A and preterminal axonal localization of 5-HT1B serotonin receptors in adult rat brain. J Comp Neurol 417: 181–194. doi:10.1002/(SICI)1096-9861(20000207)417:2<181::AID-CNE4>3.0.CO;2-A [pii].

7. Cornea-Hébert V, Riad M, Wu C, Singh SK, Descarries L (1999) Cellular and subcellular distribution of the serotonin 5-HT2A receptor in the central nervous system of adult rat. J Comp Neurol 409: 187–209.

8. Jakab RL, Goldman-Rakic PS (2000) Segregation of serotonin 5-HT2A and 5-HT3 receptors in inhibitory circuits of the primate cerebral cortex. J Comp Neurol 417: 337–348.

9. Descarries L, Cornea-Hebert V, Riad M (2006) Cellular and subcellular localization of serotonin receptors in the central nervous system. The serotonin receptors. In: Roth BL, editor. The serotonin receptors. Humana Press. pp. 277–317. doi:10.1007/978-1-59745-080-5_9.

10. Belenky MA, Pickard GE (2001) Subcellular distribution of 5-HT1b and 5-HT7 receptors in the mouse suprachiasmatic nucleus. J Comp Neurol 432: 371–388. doi:10.1002/cne.1109.

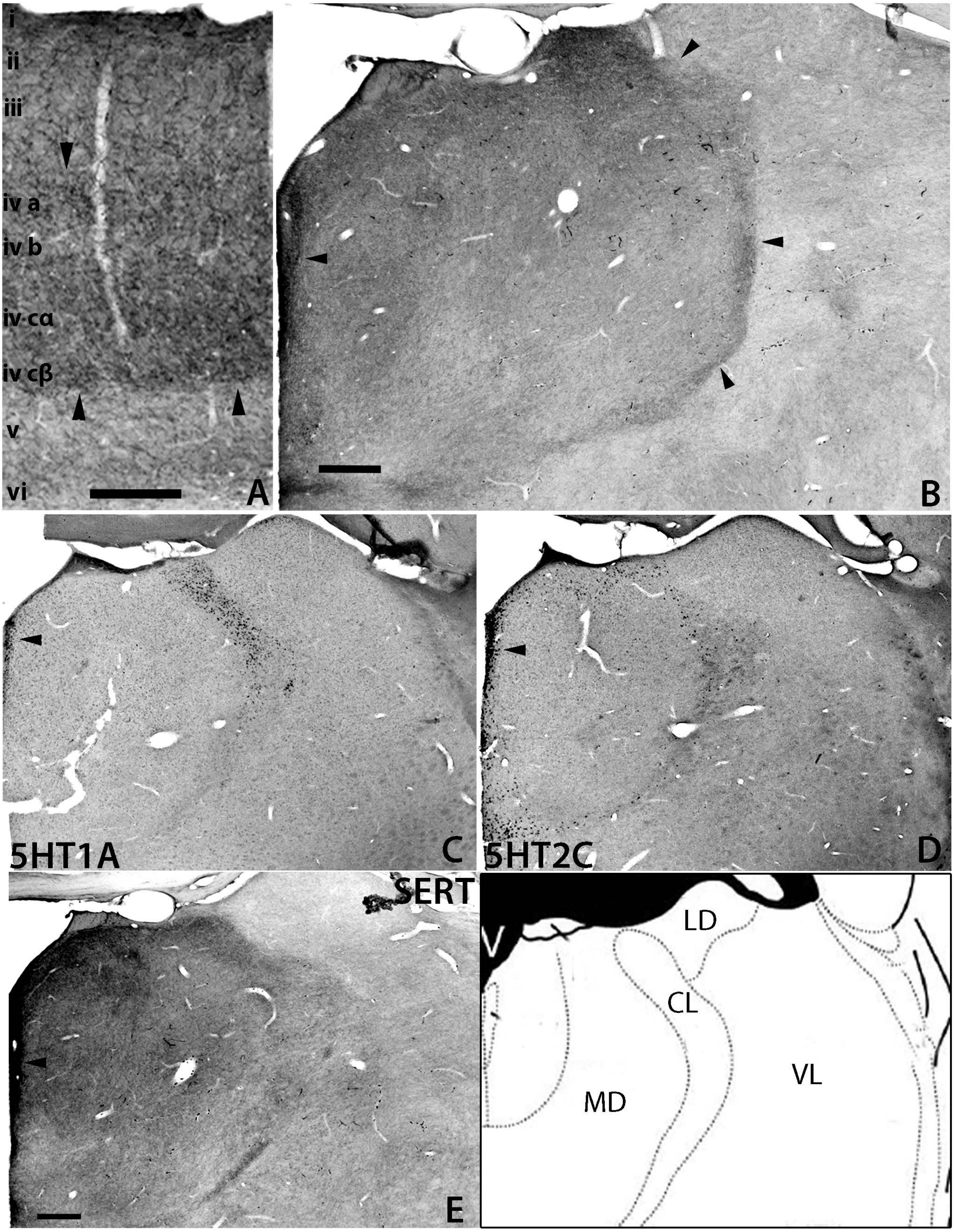

Supplementary Figure1

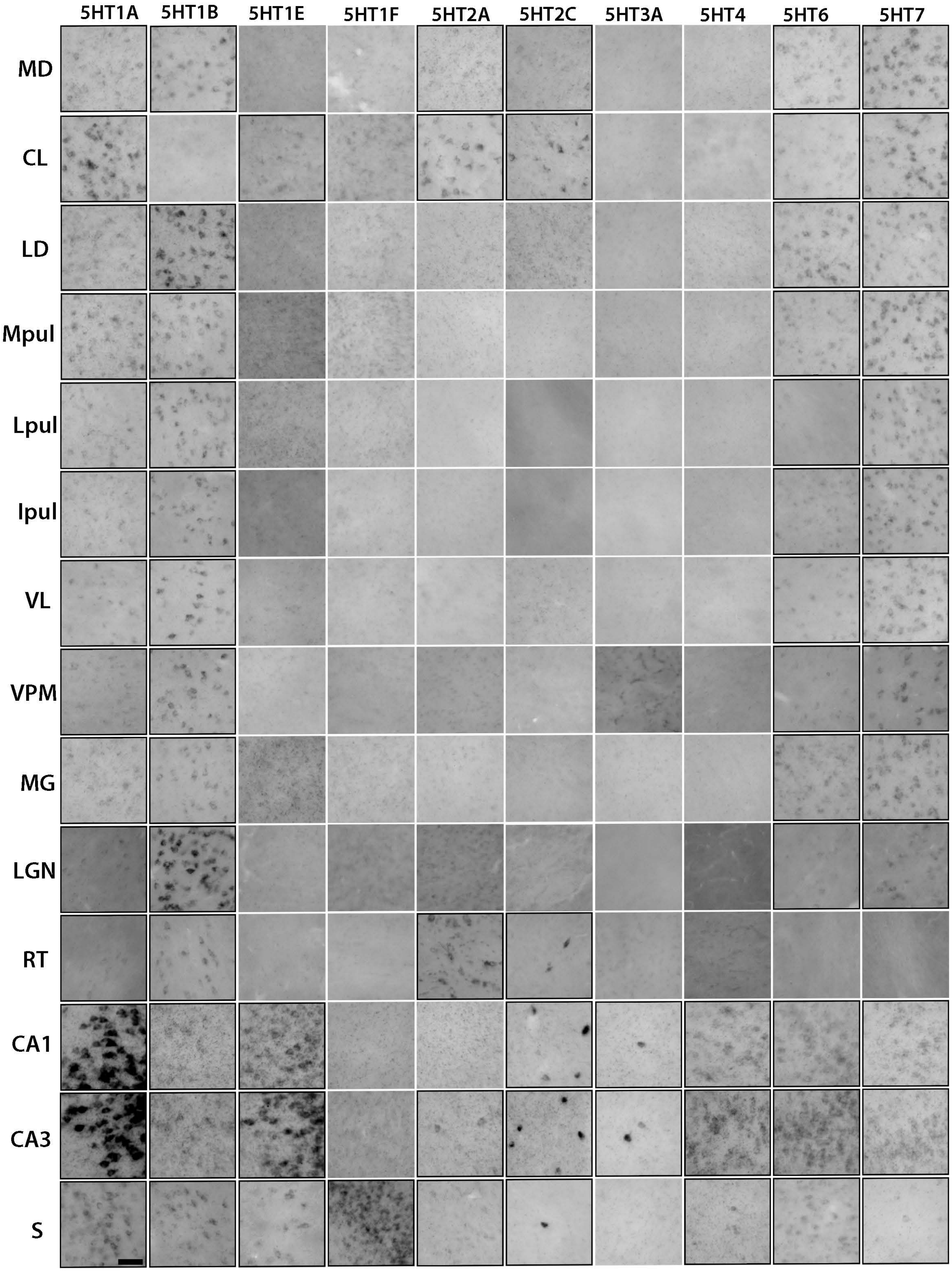

Supplementary Figure2

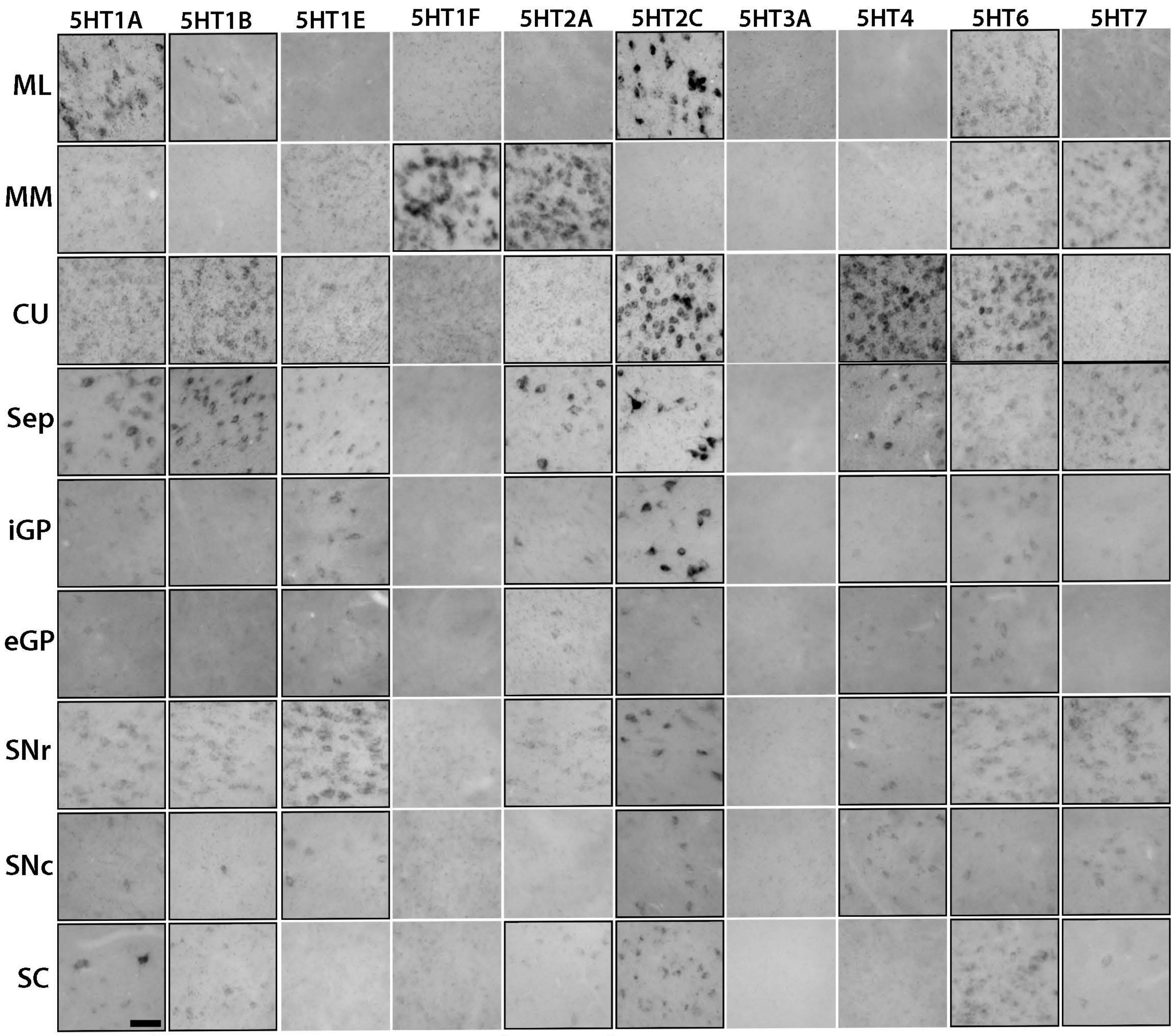

Supplementary Figure2 (Cont.)

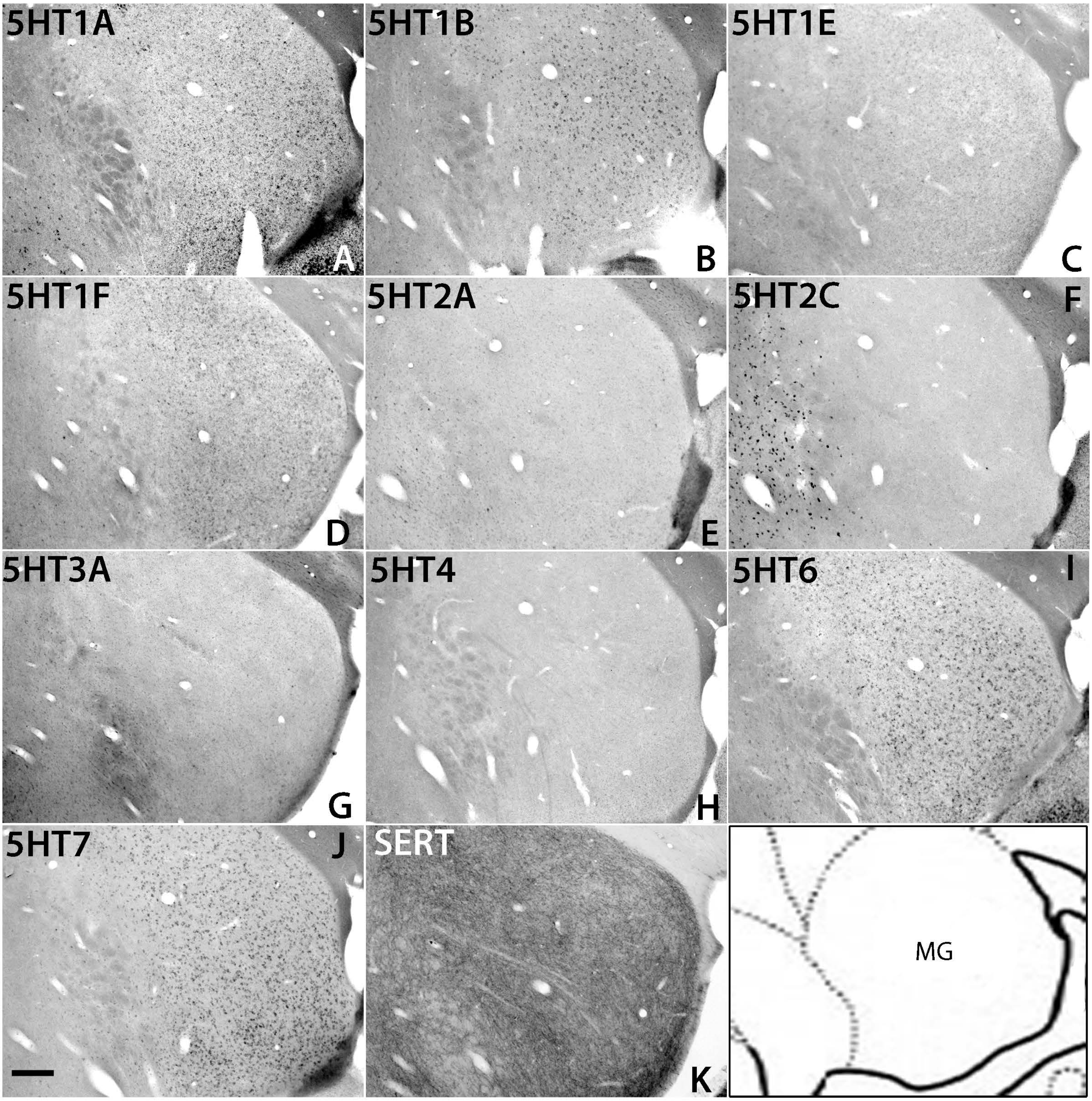

Suplementary Figure3

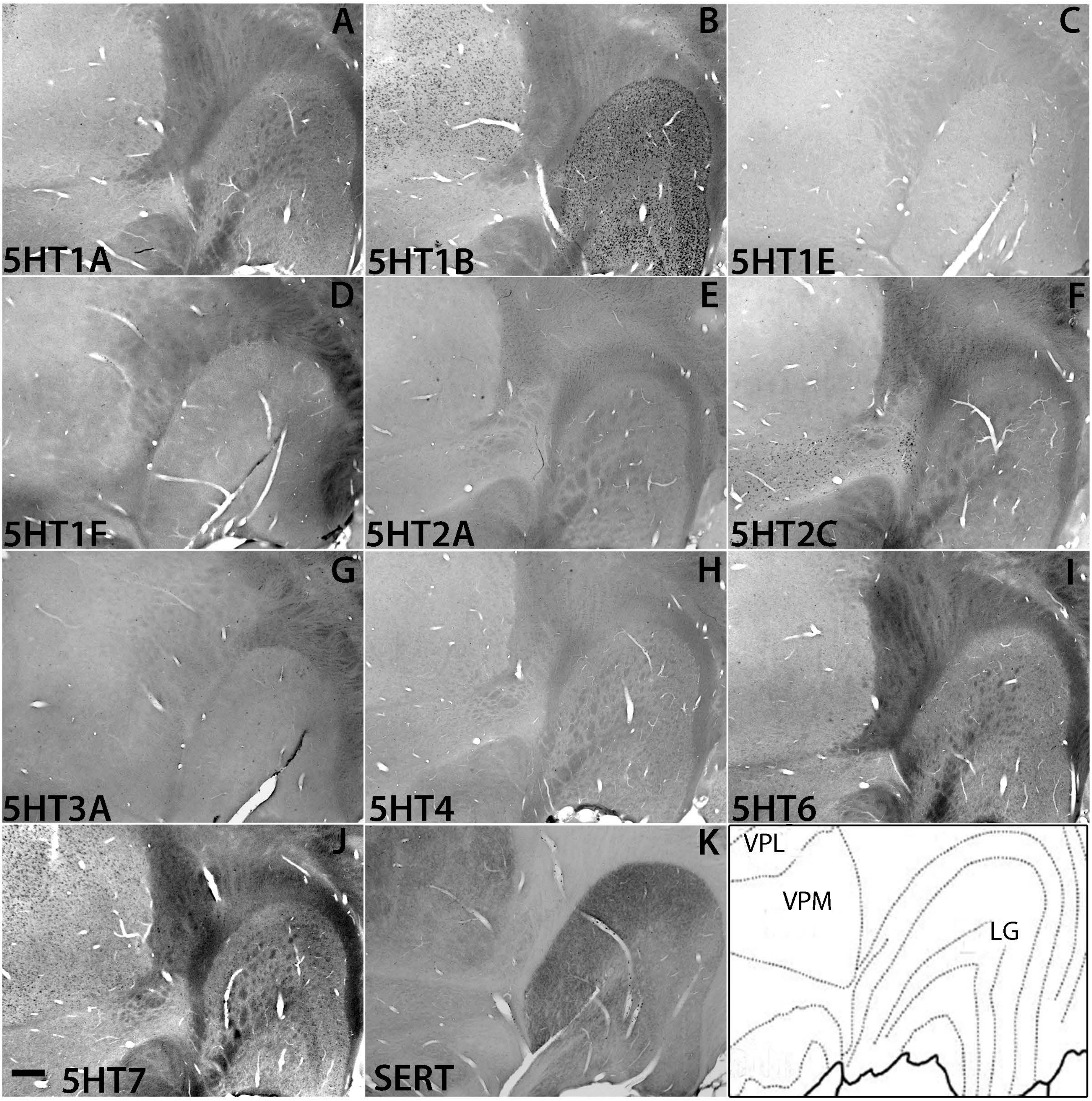

Supplementary Figure4

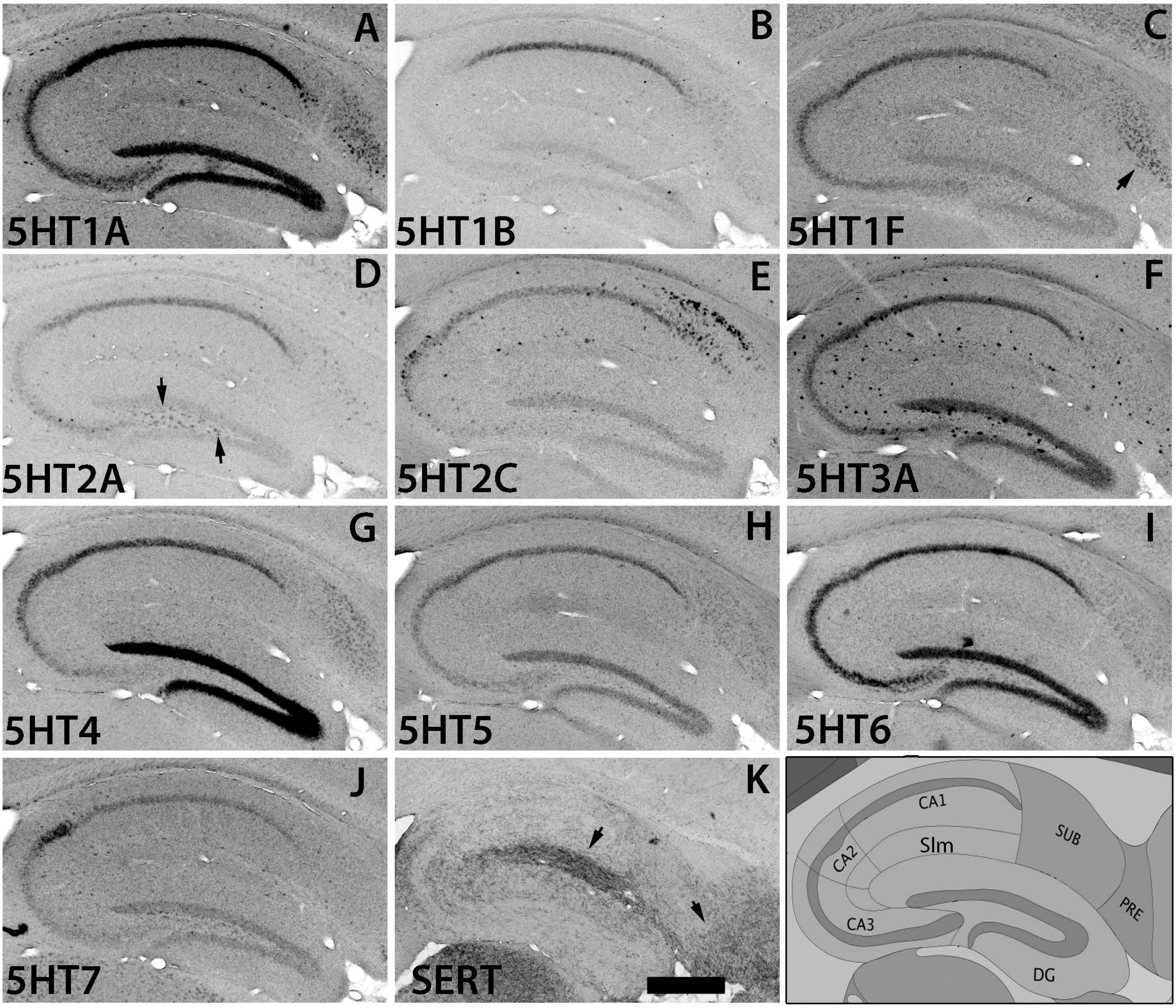

Supplementary Figure5

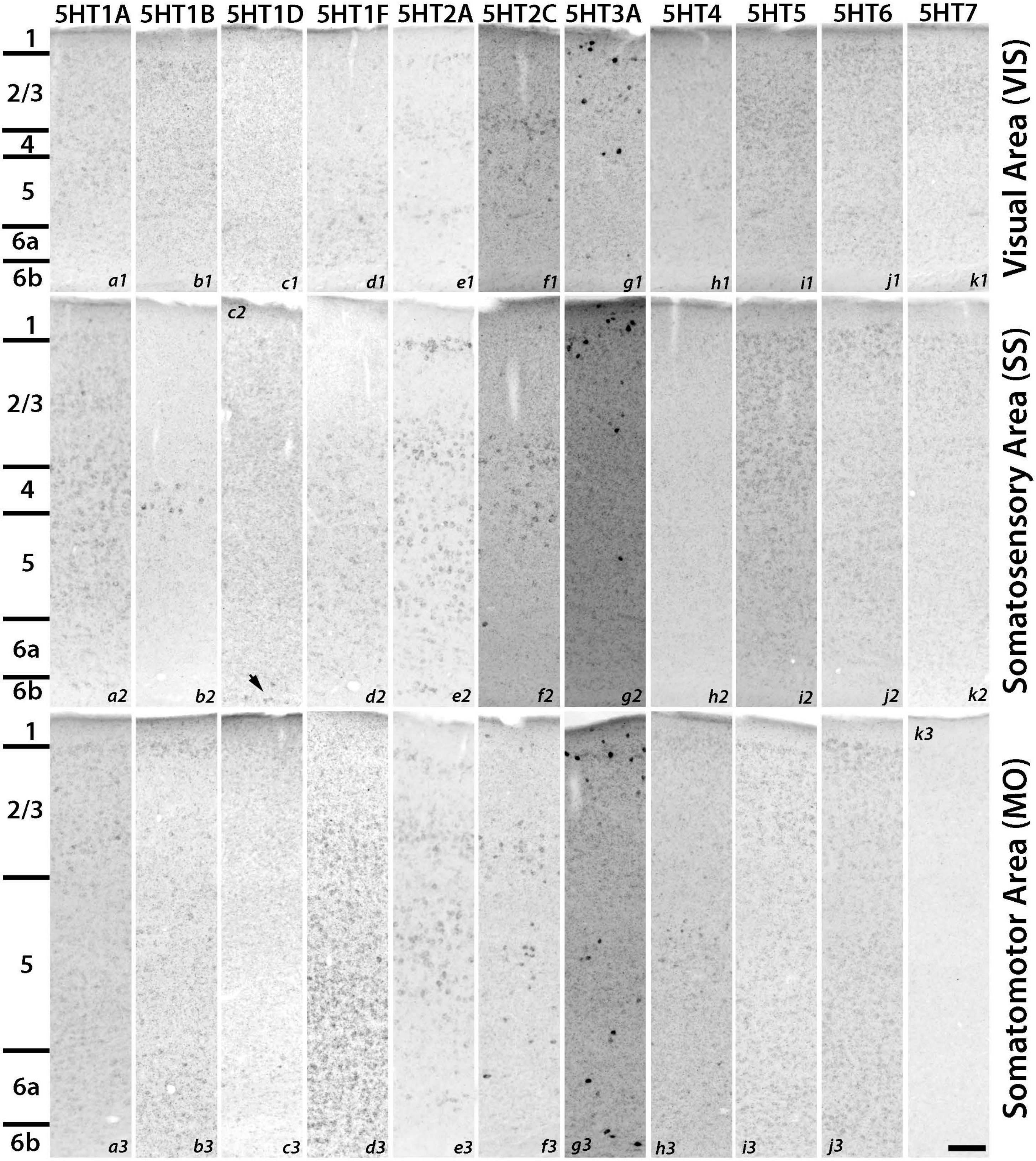

Supplementary Figure6

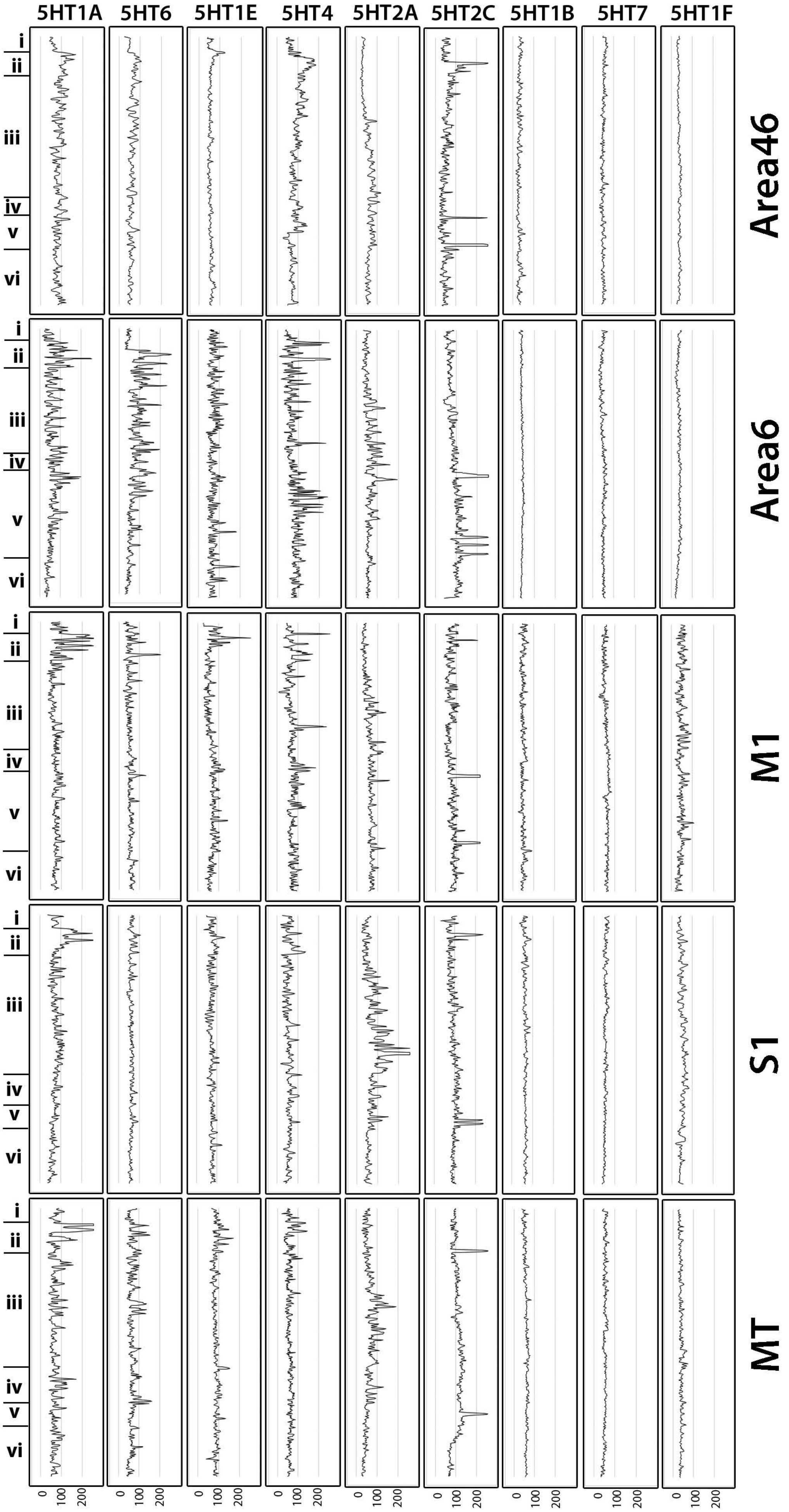

Supplementry Figure7a

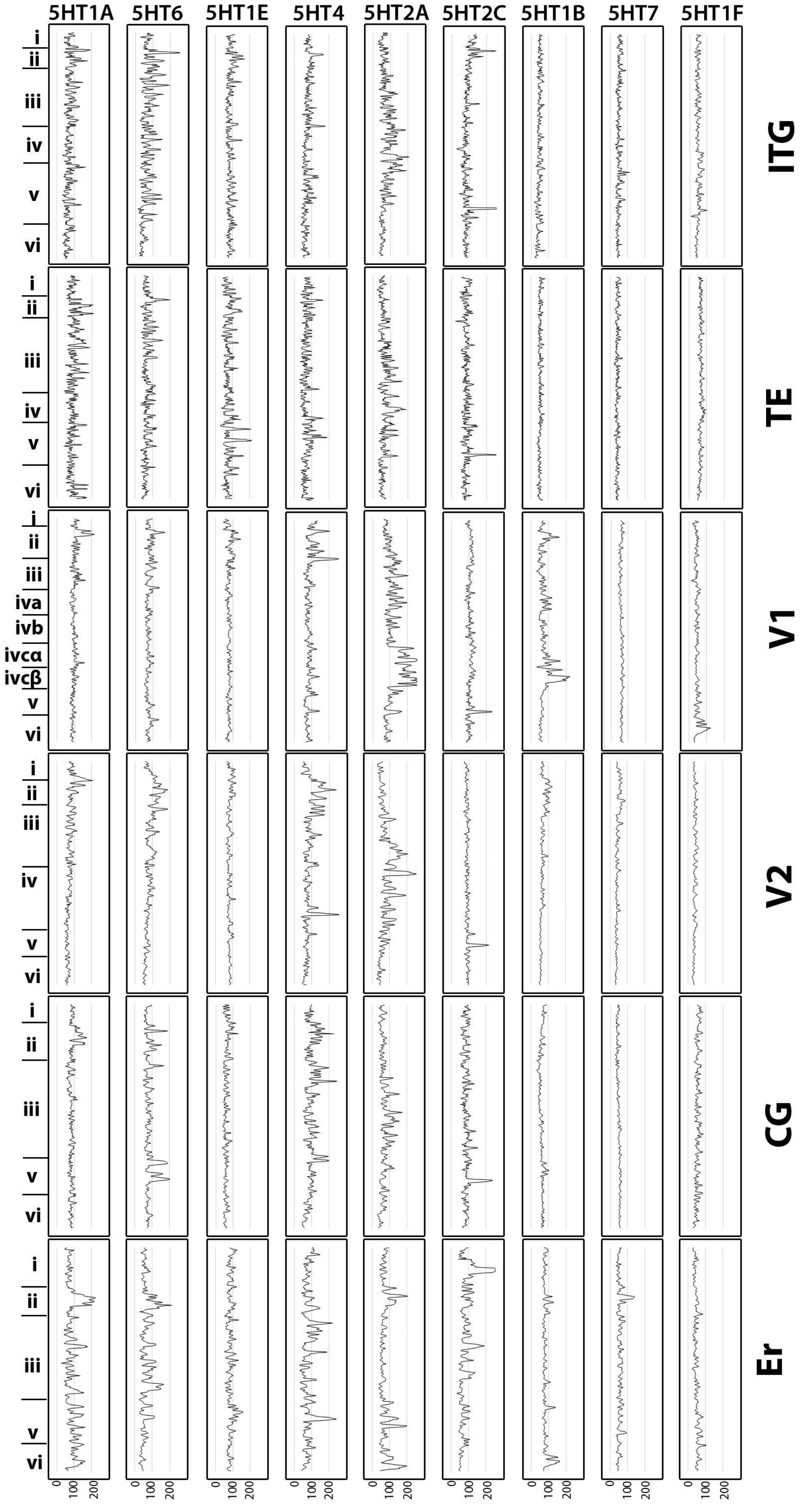

Supplementry Figure7b

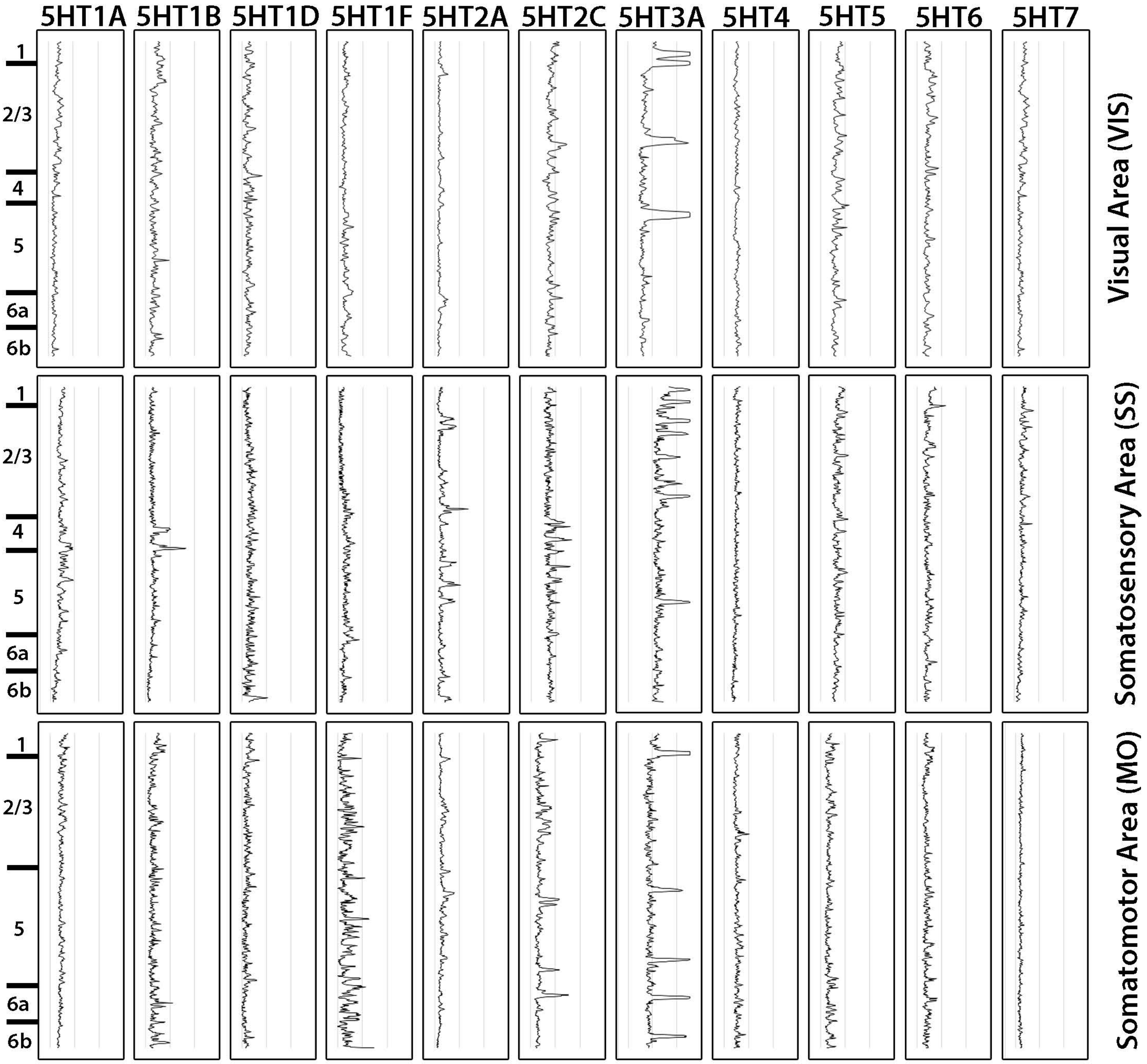

Supplementry Figure7c
