# Supplementary figures and images for: mRNA expression profile of serotonin receptor subtypes and distribution of serotonergic terminations in marmoset brain
Source: Front Neural Circuits. 2014 May 19;8:52. doi: 10.3389/fncir.2014.00052 (PMC4032978; doi:10.3389/fncir.2014.00052)

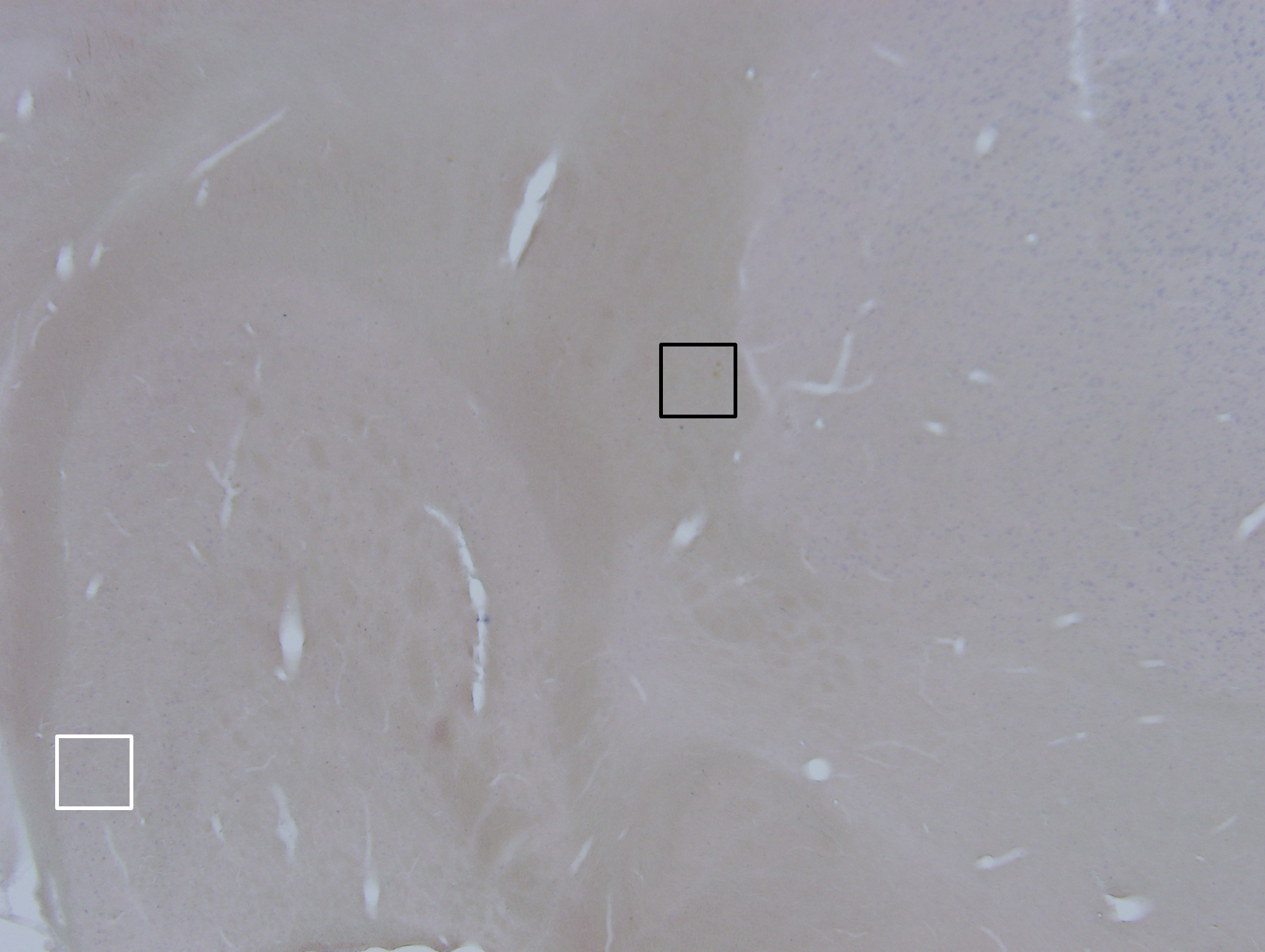

Supplement: Supplementary file 2 [file Presentation2.ZIP › Supplementary Fig8a (5HT7-LGN).tif]

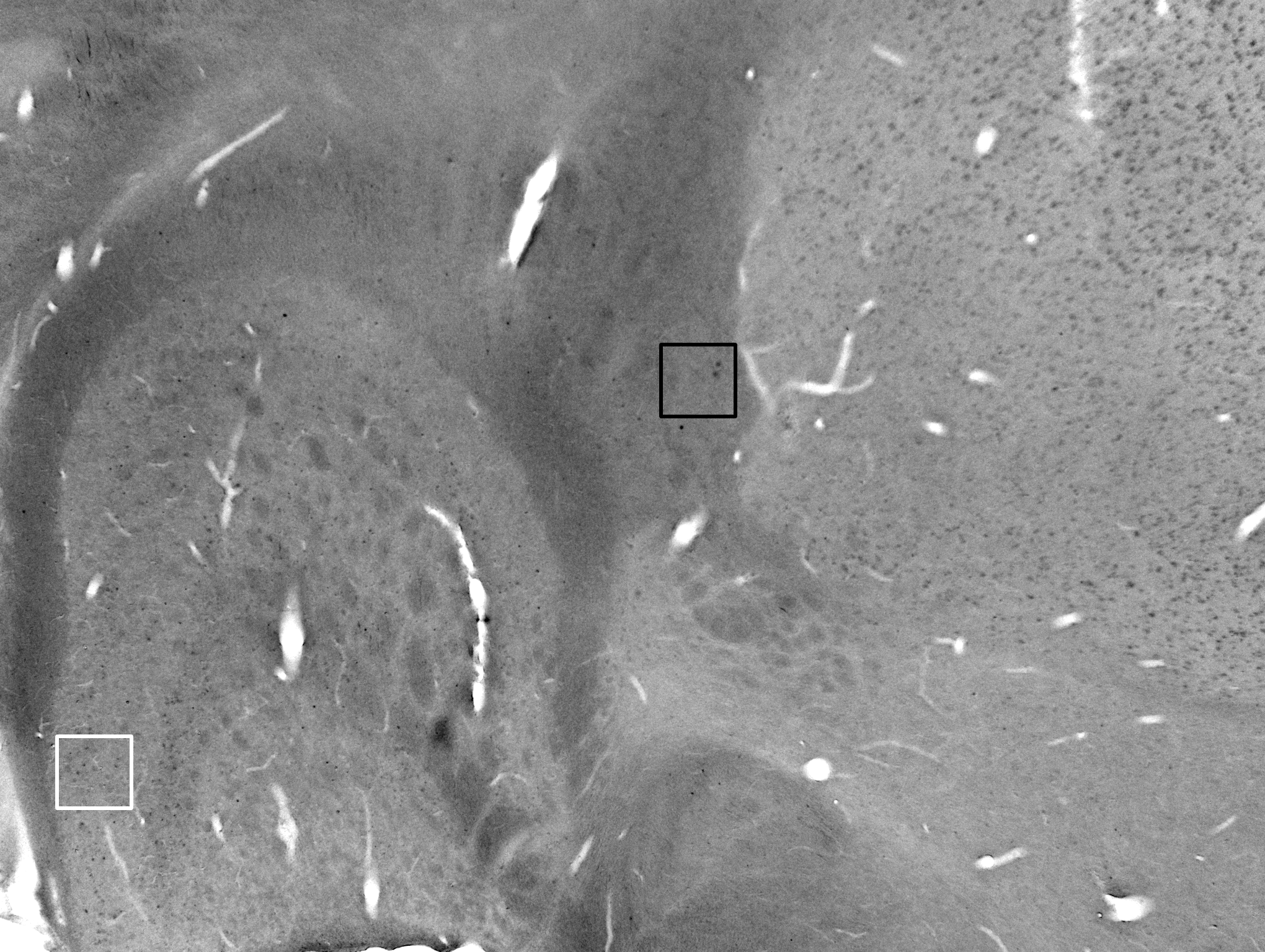

Supplement: Supplementary file 2 [file Presentation2.ZIP › Supplementary Fig8b (5HT7-LGN).tif]

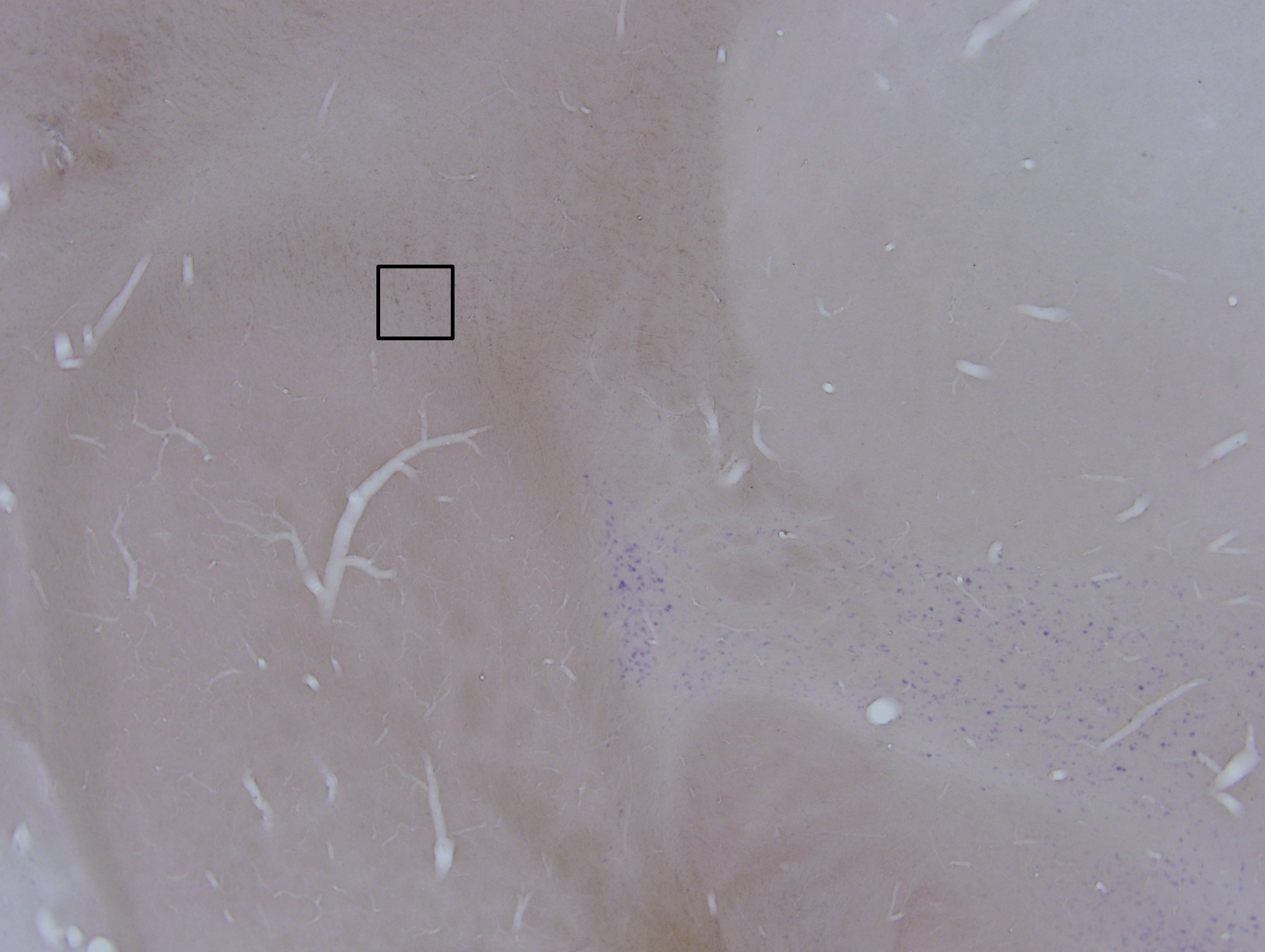

Supplement: Supplementary file 2 [file Presentation2.ZIP › Supplementary Fig8c (5HT2C-LGN).tif]

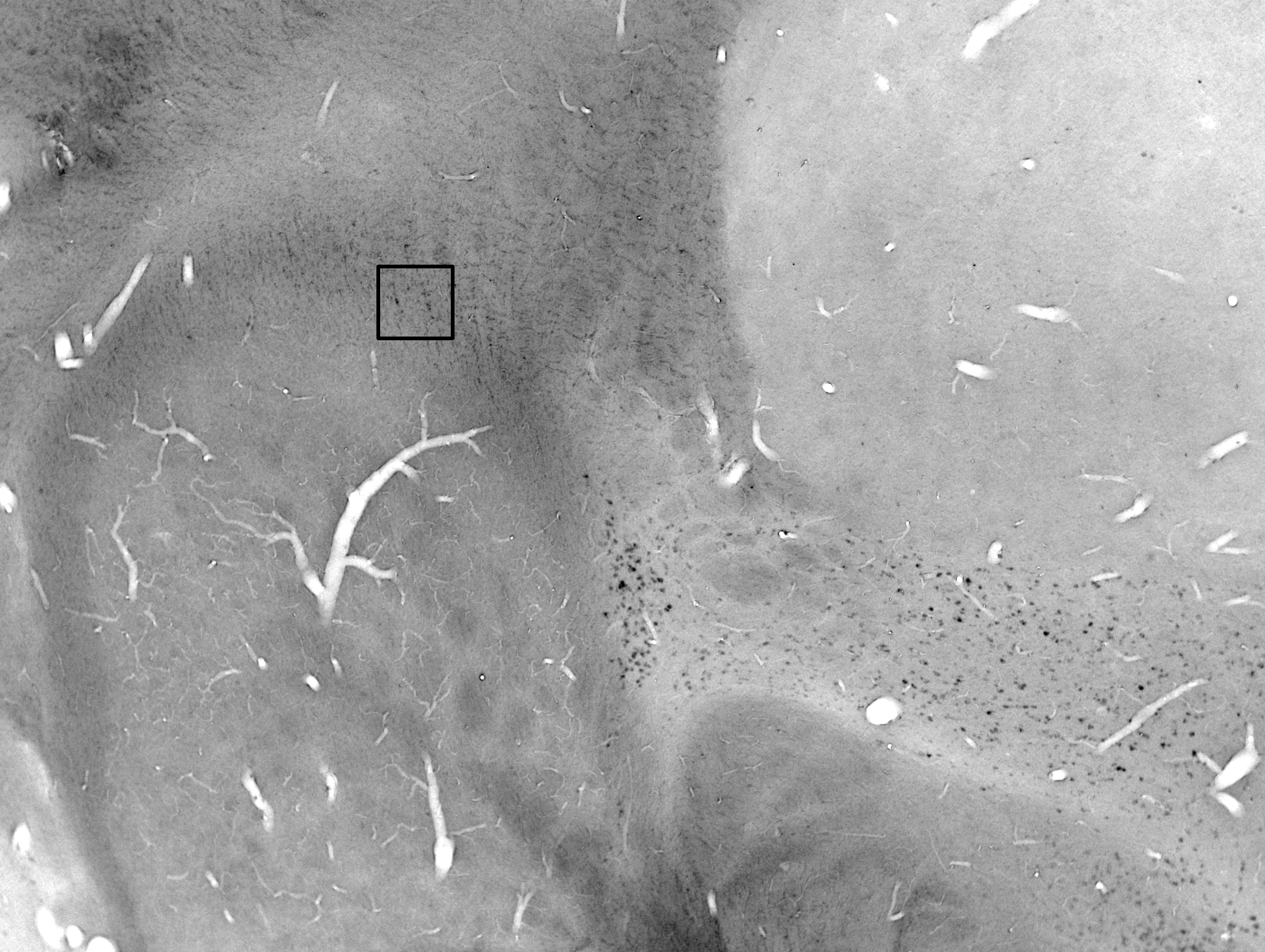

Supplement: Supplementary file 2 [file Presentation2.ZIP › Supplementary Fig8d (5HT2C-LGN).tif]
